# Upgrading of Pyrolysis Bio-Oil by Catalytic Hydrodeoxygenation, a Review Focused on Catalysts, Model Molecules, Deactivation, and Reaction Routes

**DOI:** 10.3390/molecules29184325

**Published:** 2024-09-12

**Authors:** Alejandra Carrasco Díaz, Lokmane Abdelouahed, Nicolas Brodu, Vicente Montes-Jiménez, Bechara Taouk

**Affiliations:** 1 LSPC—Laboratoire de Securité des Procédes Chimiques, INSA Rouen Normandie, UNIROUEN, Normandie Univiversity, 76000 Rouen, France; alejandra.carrasco_diaz@insa-rouen.fr (A.C.D.); lokmane.abdelouahed@insa-rouen.fr (L.A.); nicolas.brodu@univ-rouen.fr (N.B.); 2 Department of Organic and Inorganic Chemistry, University of Extremadura, 06006 Badajoz, Spain; vmontes@unex.es

**Keywords:** biomass pyrolysis, bio-oil upgrading, hydrodeoxygenation

## Abstract

Biomass can be converted into energy/fuel by different techniques, such as pyrolysis, gasification, and others. In the case of pyrolysis, biomass can be converted into a crude bio-oil around 50–75% yield. However, the direct use of this crude bio-oil is impractical due to its high content of oxygenated compounds, which provide inferior properties compared to those of fossil-derived bio-oil, such as petroleum. Consequently, bio-oil needs to be upgraded by physical processes (filtration, emulsification, among others) and/or chemical processes (esterification, cracking, hydrodeoxygenation, among others). In contrast, hydrodeoxygenation (HDO) can effectively increase the calorific value and improve the acidity and viscosity of bio-oils through reaction pathways such as cracking, decarbonylation, decarboxylation, hydrocracking, hydrodeoxygenation, and hydrogenation, where catalysts play a crucial role. This article first focuses on the general aspects of biomass, subsequent bio-oil production, its properties, and the various methods of upgrading pyrolytic bio-oil to improve its calorific value, pH, viscosity, degree of deoxygenation (DOD), and other attributes. Secondly, particular emphasis is placed on the process of converting model molecules and bio-oil via HDO using catalysts based on nickel and nickel combined with other active elements. Through these phases, readers can gain a deeper understanding of the HDO process and the reaction mechanisms involved. Finally, the different equipment used to obtain an improved HDO product from bio-oil is discussed, providing valuable insights for the practical application of this reaction in pyrolysis bio-oil production.

## 1. Introduction

**Figure 1:**
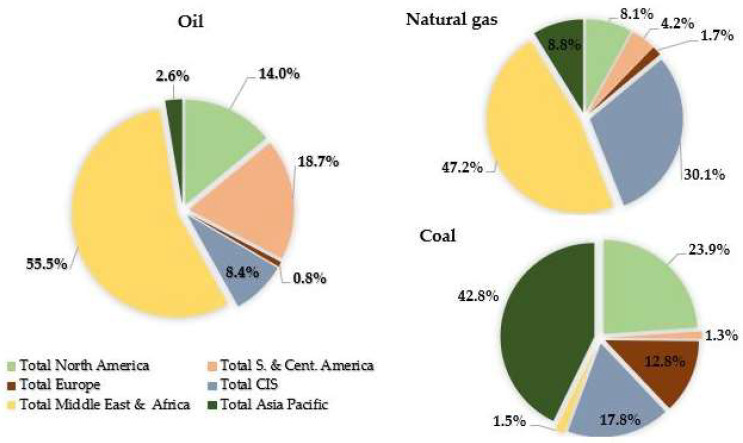
Fossil fuels reserves from [5].

The COVID-19 pandemic has disproportionately affected various communities and economies globally, and the energy sector is no exception. This crisis has highlighted the critical role of energy in maintaining homes, health systems, and digital infrastructure [1,2]. It has challenged the resilience of energy networks and has catalysed investments aimed at achieving deeper decarbonization in heating and transportation. Rising energy demand, compounded by the environmental impact of extensive fossil fuel use, significant demographic expansion (with a growth of over 1.5 million in the last two decades) [3], and the need for economic and social advancement have hastened the advancement of economical and sustainable energy solutions. Since 1973, energy consumption has doubled in developed nations, yet the demand continues to outpace supply even as reserves continue to decline (Figure 1) [3,4,5].

**Figure 2:**
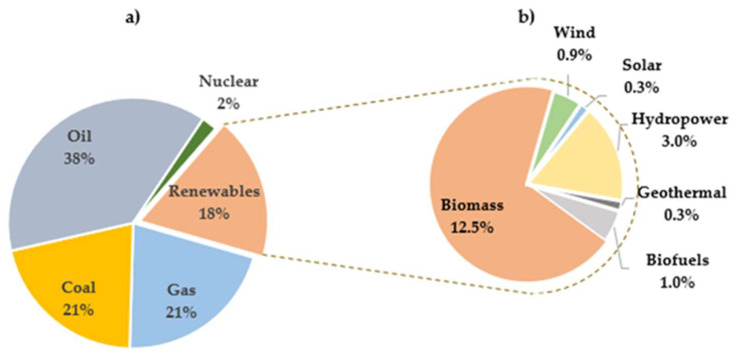
Estimated contribution of renewables in global total energy consumption in 2021, (**a**) distribution of total energy consumption, (**b**) distribution of the renewable portion of global energy consumption, from Renewable Share of Total Final Energy Consumption 2021, adapted from [3,4,5].

The inevitable depletion of fossil fuel reserves, along with the socio-political conflicts arising from their localized extraction, underscores the significant drawbacks of the global reliance on these finite resources. Energy needs are predominantly supplied through the utilization of non-renewable sources such as coal, oil, and natural gas. However, the finite nature of these resources means they could be depleted sooner than anticipated. According to data from BP’s “Statistical Review of World Energy” in July 2021 [5], the world’s current fossil fuel reserves are substantial: 244 billion tons of oil, 6642 billion cubic feet of natural gas, and 1074 billion tons of coal. These numbers may appear significant initially, but projections suggest that natural gas reserves could be exhausted by 2068, with oil reserves depleted by 2065. As shown in Figure 2, coal is allocated in more regions compared to natural gas and particularly oil, of which 75% of reserves are held by OPEC nations, predominantly located in the Middle East [5].

In 2021, renewable energies contributed 18% to primary energy production, 8% larger from 2006, as indicated in Figure 2a. Among renewables, approximately 5% stemmed from hydropower, wind, solar, and biofuels. Biomass, mainly used for consumption, represented about 10% of total renewable energy, with a smaller fraction allocated to energy, fuels, and chemical feedstocks. Hydropower accounted for 3% of global final energy consumption, while other renewables, including wind, biofuels, and geothermal sources, constituted 2.0% in 2021. This growth was particularly evident in developed countries, as depicted in Figure 2b of the Supply—Key World Energy Statistics 2021—Analysis [6]. This article first focuses on the general aspects of biomass, subsequent bio-oil production, its properties, and the various methods of upgrading pyrolytic bio-oil to improve its calorific value, pH, viscosity, degree of deoxygenation (DOD), and other attributes. Secondly, particular emphasis is placed on the process of converting model molecules and bio-oil via HDO using catalysts based on nickel and nickel combined with other active elements. Through these phases, readers can gain a deeper understanding of the HDO process and the reaction mechanisms involved. Finally, the different equipment used to obtain an improved HDO product from bio-oil is discussed, providing valuable insights for the practical application of this reaction in pyrolysis bio-oil production.

## 2. Biomass as Renewable Energy Source

Biomass can be defined as organic matter derived from plants or animals, which is produced through biological process, whether induced or spontaneous. This material can be broadly categorized into non-lignocellulosic or lignocellulosic forms, including woody, herbaceous, aquatic residues, manure, agricultural sub-products, and more [7]. Its classification as a renewable energy source stems from its ability to regenerate at a rate that exceeds its consumption [8]. The energy contained in biomass originates from the photosynthesis process of plants. Through valorization techniques like anaerobic digestion, combustion, pyrolysis, and gasification, this energy can be released artificially.

**Figure 3:**
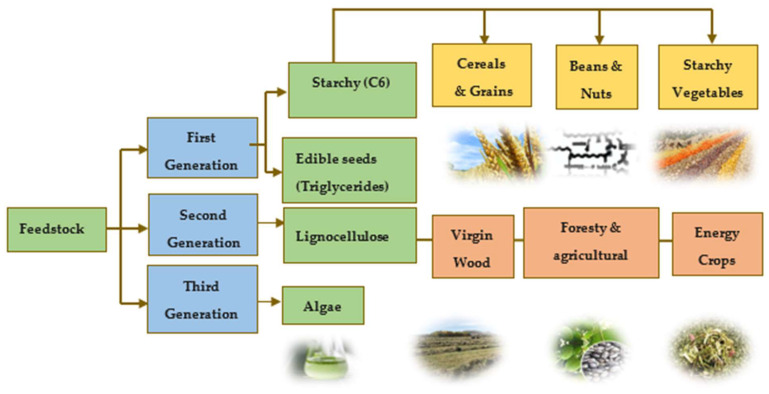
Classification of biomass feedstock in three generations, adapted from [9].

From the energy aspect, one key advantage of using biomass is its capacity not to upsurge CO_2_ emissions. Unlike fossil fuels, the emissions from biomass combustion derive from carbon that was recently extracted from the atmosphere during the plant’s growth cycle. This means that the release of CO_2_ does not disrupt the balance of atmospheric carbon concentration, thus mitigating the greenhouse effect. However, there is ongoing debate regarding the comprehensive assessment of energy consumption throughout the production process and its impact on greenhouse gas emissions. To address this, employing analytical methodologies like life cycle assessments and carbon footprint analyses becomes imperative in evaluating the environmental sustainability of various biofuel production techniques. Biomass is classified into different generations according to the various types of feedstocks used Figure 3. Biomass classifications are alterable and depend on their variety, composition, and quantity [9].

The chemical composition significantly influences the reactivity of biomass, so it must be well known in order to optimize the transformation process, both for obtaining biofuels and chemical products, with high selectivity and efficiency [10].

### Fast Pyrolysis: Pyrolytic Bio-Oil, Composition and Properties

Fast pyrolysis is a thermal decomposition process where biomass is rapidly heated to high temperatures without oxygen. This process targets the breakdown of hemicellulose, cellulose and lignin. It occurs in a temperature range of 300–700 °C at a faster heating rate of 10–200 °C/s, a short residence time between 0.5 and 10 s, and a feedstock with a fine particle size (<1 mm) [11], this method yields char, a solid residue with carbon, along with volatiles and gases [12]. After cooling to room temperature, some of these volatiles condense into a tar or pyrolytic bio-oil, around 30–40 wt% of the original biomass weight and containing diverse organic compounds with approximately 25 wt% water content sourced from both biomass moisture and decomposition [13].

In general, organics compounds are classified into aldehydes and ketones (~15.0 wt%), acids (~35.0 wt%), phenols (~20.0 wt%), alcohols (~8.0 wt%), furans (~2.75 wt%) and others (~20.0 wt%) [14,15]. All these oxygenated compounds are challenging for direct applications of bio-oil as a result of its physical and chemical properties, such as high water content, high acidity, high proportion of oxygen and low calorific value (LHV: 15–20 MJ/kg), almost a half compared to petroleum as shown in Table 1 [16,17].

**Figure 4:**
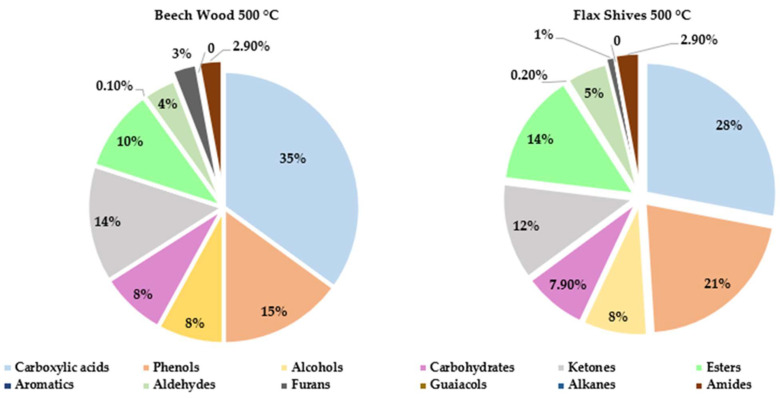
Chemical composition of bio-oils from pyrolysis of two different feedstocks, beech wood and Flax shives, at 500 °C, adapted from [15,22].

Therefore, the bio-oil must be upgraded by physical or chemical methods. The specific distribution of chemical families in pyrolytic beechwood and flax shives oils obtained at 500 °C have been found in the literature and can be seen in Figure 4. It is clear that acids are the primary chemical family in the pyrolytic bio-oil followed by phenols, ke-tones, al-cohols and anhydrosugars [15].

## 3. Bio-Oil Upgrading Methods

**Figure 5:**
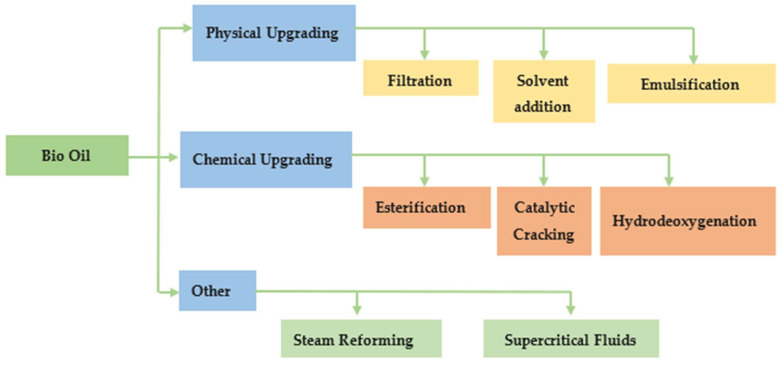
Upgrading methods of bio-oil, adapted from [21,24,25].

As discussed above the direct application of Pyrolitic bio-oil as petroleum-based fuel is inconceivable [21,23]. The limitations of bio-oil can be removed by different upgrading methods; however, intensive research has been done so far on these methods. Here, recent developments in bio-oil upgrading methods are presented. Bio-oil upgradation can be classified as the following three types, physical, chemical, and others, shown in Figure 5.

### 3.1. Emulsification

Emulsification improves the low solubility of bio-oil in hydrocarbon fuels by mixing it with diesel and surfactants, leading to a more stable mixture and improving fuel properties [21,25]. The emulsification method depends on the hydrophilic–lipophilic balance (HLB) of surfactants, which can either lipophilic (4–8) or hydrophilic (9–15) [21,23]. Successful emulsification needs immiscible bio-oil and diesel, surfactants to aid mixing, and stirring required for dispersion [21,23]. Bio-oil emulsification with diesel may be difficult because of phase separation, and it requires precise proportions of bio-oil (10–50%), diesel (50–90%), and surfactants (1–10%) [26,27]. This process increases the calorific value, pH, and stability of the bio-oil [26]. Ikura et al. [27] reported that increased bio-oil content increased the viscosity of the emulsion, yielding optimal stability at 10–20 wt% of bio-oil and 4–6 wt% of surfactant. The addition of alcohols such as methanol, ethanol, and n-butanol as co-surfactants improved stability [28]. Farooq et al. [27] demonstrated that the use of Span 80 and Tween 60 increased the heating value of ether extracted emulsified bio-oil (EEO) up to 44 MJ/kg, maintaining stability for 40 days. In addition, emulsifiers such as Span 60, Span 80, Tween 80, and lecithin are common in bio-oil emulsification and sometimes need to be mixed to obtain optimal HLB values, although differences in thermal stability must be considered [26,27,29,30,31]. Also, emulsified bio-oil may act as a lubricant, with increased bio-oil content increasing lubrication [32].

### 3.2. Esterification

Esterification transforms the carboxylic acids in bio-oils into more stable esters by reacting them with alcohols and often using zeolite-supported catalysts, leading to a fuel suitable for combustion engines [33]. This process reduces the acid number, density and water content of bio-oil, while enhancing its calorific value [34]. Usually, bio-oil is esterified using alcohols such as ethanol or methanol at 50–60 °C for 1–5 h, which reduces the viscosity and increases the calorific value. Methanol is preferable due to its efficiency as a solvent [34,35]. Prasertpong et al. reported catalytic esterification with ethanol using 12-tungsto-silicic acid reduced acidity by 38–85% and increased calorific values by about 32% [36,37]. Despite these improvements, the calorific value of the improved bio-oil of 17.6 and 23.2 MJ/kg suggests further improvement is needed [36].

### 3.3. Solvent Addition

Bio-oil, which contains active oxygen compounds and a high viscosity, presents challenges as a transport fuel due to increasing viscosity over time caused by condensation and polymerization [21]. Solvent additions such as methanol, ethanol, ethyl acetate, and acetone help to homogenize the blend, reduce the viscosity and density, and increase the calorific value [38]. In particular, alcohols avoid phase separation by increasing the solubility of hydrophobic compounds, resulting in a top layer of enriched bio-oil [39]. Feng et al. researched phenol hydrogenation over palladium on activated carbon catalysts, discovering that the polarity of the solvent affected the conversion of phenol. Phenol was converted fully to hexane and water at 250 °C, but only partially to methanol or ethanol, highlighting the synergistic effects of various factors on phenol hydrogenation. Both water and hexane were identified as effective solvents for upgrading the pyrolysis bio-oil, enhancing phenol conversion [40].

### 3.4. Steam Reforming (SR)

Steam reforming turns hydrocarbons into CO and H_2_ by reacting them with steam at high temperatures, also involving water–gas shift (WGS) and methanation reactions, influenced by operational conditions [41]. H_2_ production via steam reforming from bio-oil requires a high-temperature reactor [42]. Some studies have explored the conversion of bio-oil to syngas using fluidized bed reactors and Ni catalysts, simulating methane steam reforming at 600–900 °C [43]. Bio-oil, represented as C_a_H_b_O_c_ and xH_2_O, undergoes several reactions: steam reforming, WGS catalytic cracking, Boudouard reaction, and methanation [44]. These reactions together produce H_2_, CO_2_, CO, CH_4_, and solid carbon. Transition metals such as Ni, Co, and Cu are preferred as catalysts due to their lower cost compared to noble metals such as Rh, Pt, and Ru [45]. Yan Ding et al. proved the effectiveness of NiO/NaF catalysts for hydrogen production on various oxygen-containing volatile organic compounds (OVOCs), showing high H_2_ selectivity for formic acid, formaldehyde, and methanol at specific temperatures [43].

### 3.5. Catalytic Cracking (Zeolite)

Catalytic cracking involves treating the steam with a suitable catalyst to break down the organic compounds and produce an oxygen-free product [44]. A variety of catalysts, such as solid acids, zeolites, alumina, and metal oxides, are used for this purpose, either integrated in the biomass or supplied separately. Catalysts based on zeolite, particularly HZSM-5 [46,47], are notable for their porous structure and efficiency in biomass conversion, particularly for aromatics production, due to their acidity, thermal stability, and suitable pore size. Studies by Kurnia et al. [48] proved that CuNi/zeolite, Cu/zeolite, and Ni/zeolite catalysts significantly increased the bio-oil yield compared to pyrolysis without catalysts. Catalyst structure and acidity influenced bio-oil composition, affecting the product distribution, coke formation, and deoxygenation rate. In addition, zeolites with high alumina content could turn oxygenated compounds into aromatic hydrocarbons, with catalyst regeneration possible by calcination. Catalysts based on alkali metals and Ni have been effective in reducing tar formation during pyrolysis, although carbon deposition in the catalyst structure can hinder activity [48,49,50,51,52].

### 3.6. Super Critical Fluids

The SCF upgrading method improves the calorific value and reduces the viscosity of bio-oil using the unique properties of supercritical states to dissolve typically insoluble materials in liquid or gaseous solvents [52]. SCFs are used in bio-oil production, especially in the hydrothermal liquefaction of biomass, together with catalysts and solvents such as ethanol, methanol, CO_2_, and water [53,54]. For example, Duan et al. [52] reported an enhanced calorific value of algal bio-oil during gasification in supercritical water with a Ru/C-Rh/C catalyst. Similarly, Kazmi et al. documented the complete transformation of carboxylic acids to esters in supercritical ethanol [55]. Lee et al. demonstrated improved bio-oil quality in supercritical ethanol using Ni-based catalysts [56], while Prajitno et al. achieved remarkable improvements in bio-oil properties in supercritical ethanol without external catalysts [57]. Furthermore, Zhang et al. found that combining hydrodeoxygenation with supercritical fluid systems significantly increased the calorific value of bio-oil and improved deoxygenation levels [58]. These studies highlight the potential of supercritical fluid systems in bio-oil upgrading, offering avenues to improve biofuel properties and energy efficiency. Table 2 summarizes the essentials of the comparison of the different methods of improving bio-oils.

## 4. Hydrodeoxygenation

Considerable research efforts have been dedicated to enhancing the quality and utilization efficiency of pyrolysis oil through methods such as hydrodeoxygenation (HDO), emulsification, hydrocracking, reforming for hydrogen production, and others. Among these techniques, HDO stands out due to its comparative advantages over the latter three methods. While emulsification can be relatively corrosive, hydrocracking often yields low results, and hydrogen reforming is both expensive and still in its developmental stages. In contrast, HDO offers a promising solution by effectively saturating aromatic components and alkenes, thereby increasing the calorific value of the bio-oil through an elevated H/C ratio and reducing the O/C ratio in organic compounds, where oxygen is removed in the form of water (which is environmentally friendly, unlike CO_2_ in the case of deoxygenation) [25].

**Figure 6:**
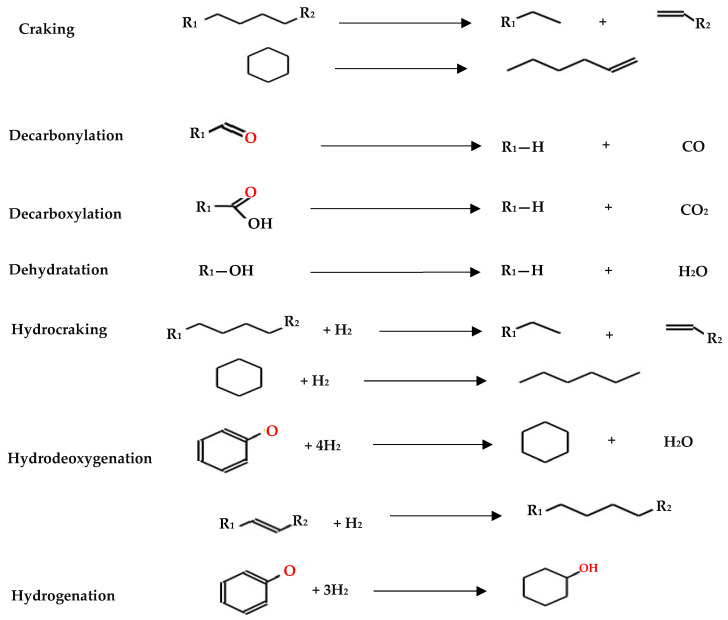
Typical reactions involved in HDO, adapted from [36,58,63,64,65].

This underscores the significance of HDO as a valuable approach for improving the quality and energy density of pyrolysis oil, presenting opportunities for more efficient utilization of biofuels. Selecting the right catalyst is crucial for an efficient hydrodeoxygenation process. Lignin-based model compounds are commonly used for testing catalytic performance due to the complex structure of lignin-derived bio-oil, which contains many different molecules [36,63]. During HDO, various reaction pathways such as decarbonylation, hydrogenation, cracking, and hydrocracking influence product selectivity (Figure 6), determining the desirability of the outcomes. Hydrodeoxygenation and hydrogenation are particularly important because they are directly linked to producing transportation fuels.

## 5. Supported Catalyst for the HDO

Catalyst supports, also known as carriers, play crucial roles in the hydrodeoxygenation (HDO) reaction. Apart from dispersing and stabilizing the promoter and active phases, they also offer secondary functional sites, such as Brønsted and Lewis acid sites, which are essential for the deoxygenation reaction [25]. A wide array of noble, non-noble, and transition metal catalysts, in various forms like sulfide, oxide, carbide, and phosphide, have been assessed for their HDO activity.

In HDO, hydrogenation occurs only at metallic sites, while acidic sites are required for dehydration, deoxygenation, and hydrogenolysis. Hence, bifunctional catalysts are highly sought after in HDO applications [66,67]. The stability of metal under operating condition is a critical parameter in catalyst design, and the choice of support is based on its acid–base and structural–morphological properties. Notably, the support material significantly impacts the overall reactivity of the catalyst. Aspects such as surface area, mesoporous–microporous volume, acid–base properties, and electronic interactions with metals strongly influence HDO efficiency. Additionally, tuning of the catalyst composition plays a vital role in minimizing its deactivation. Therefore, meticulous catalyst design is vital in bio-oil upgrading by HDO [68].

### 5.1. Sulfide Catalysts

CoMoS_2_ and NiMoS_2_ are widely used in both conventional hydrotreatments and also HDO reactions. In these processes, either Cobalt (Co) or Nickel (Ni) serves as a promoter, facilitating electron transfer to the Molybdenum (Mo) atom. This electron transfer weakens the bond between Mo and S, resulting in the creation of an S vacancy site, which is known to be an active site during the HDO process [13].

In the treatment of petroleum crude oil, CoMoS_2_ [69] and NiMoS_2_ [58] can persist in sulfide form, owing to the presence of H_2_ and hydrogen sulfide (H_2_S) in the reactor. These components are typically derived directly from the thiols present in petroleum oil. However, in the case of biomass, which generally has limited or zero sulfur content, maintaining sulfide form is challenging. Without a sulfur source added to the system, CoMoS_2_ and NiMoS_2_ are often converted into their respective oxide forms [36]. Table 3 presents the HDO of various feedstocks over sulfide catalysts.

While the addition of extra sulfur could prevent this conversion, it poses risks such as catalyst poisoning during post-processing and sulfur oxide (SO_x_) emissions upon combustion. Thus, finding a balance between maintaining catalyst activity and avoiding environmental harm is crucial in these processes.

**Figure 7:**
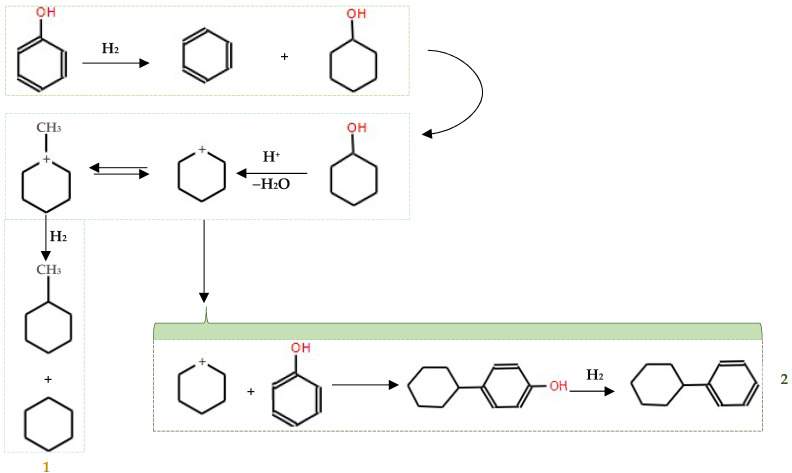
A possible reaction mechanism for the HDO of phenol over MoS_2_, adapted from [77].

Liu et al. [78] reported that 99.3% oxygen removal efficiency from the *p*-cresol at 300 °C was achieved using a dispersed unsupported MoS_2_ catalyst. All the hydrothermally synthesized MoS_2_ catalysts showed much higher activity in the HDO of *p*-cresol than the commercial MoS_2_ sample. It seems that the catalytic activity of MoS_2_ is strongly related to the surface area and morphology [77] and the high catalytic activity of molybdenum sulfide in the catalytic HDO of phenol at 450 °C and 5 MPa H_2_. The reaction mechanism for the HDO of phenol over MoS_2_ was proposed by Yang et al. [77]. They found that the hydrogenolysis of phenol produced benzene, with cyclohexanol serving as an intermediate. This intermediate can subsequently be converted into cyclohexane, methylcyclopentane, and C_12_ hydrocarbons, as depicted in Figure 7.

### 5.2. Oxide Catalysts

Previous research has highlighted the effectiveness of oxide catalysts like Mo, Ni, W, and V in catalyzing the HDO reaction. These catalysts operate on a basic mechanism where a balance between low hydrogen (H_2_) pressure prevents the conversion of active species into inactive ones, while high H_2_ pressure is crucial for preventing coke formation. The catalytic activity of oxides in HDO primarily relies on acidic sites, with Lewis acidity playing a significant role in the initial chemisorption stage. The presence of oxygenated compounds allows chemisorption, and the availability of acidic sites is influenced by Brønsted acidity [79]. In a study by Mathew et al. [80], bimetallic Pt-WO_x_/Al_2_O_3_ catalysts with varying WO_x_ loadings were examined for their effectiveness in converting benzyl alcohol to toluene through HDO. It was found that moderate WO_x_ loadings resulted in the most significant improvements in HDO activity and selectivity.

It has been reported previously in the literature that oxide catalysts, such as Mo, Ni, W, and V, exhibit significant catalytic activity in the HDO reaction. Based on the basic mechanism of oxide catalysts, a low H_2_ pressure is needed to limit the transformation of active species into inactive species; however, a high pressure of H_2_ is a necessity to avoid coke formation during the HDO process. Generally, the catalytic activity of oxides in the HDO mainly depends on the acidic sites. At the initial chemisorption stage, the Lewis acidity is the key factor, the oxygen lone pair of the oxygenated compounds can be chemisorbed, and the availability of acidic sites on the oxide catalyst is affected by the Brønsted acidity [79]. Mathew Jon et al. [80] studied bimetallic Pt-WO_x_/Al_2_O_3_ catalysts at varying WO_x_ loadings, which were tested for the HDO of benzyl alcohol to toluene. Moderate WO_x_ loadings led to the greatest enhancements in HDO activity and selectivity.

### 5.3. Transition Metal Catalysts

Transition metal catalysts, including noble metals and nickel (Pt, Pd, Ru Rh, and Ni), favor HDO and hydrogenation reactions, and their reaction rate is proportional to H_2_ pressure. Compared with sulfide-based catalysts, it is not necessary to have an additional source of sulfur to maintain the active form [81]. The main disadvantage of transition metal catalysts is related to their high sensitivity to sulfur; therefore, the removal of sulfur-containing compounds from the bio-oil is required prior to HDO treatment [79].

In a previous study by Gutierrez et al. [69], it was observed that the catalytic activity in HDO of guaiacol in hexadecane at 100 °C and 80 bars H_2_ was influenced by the catalytic metals as follows: Rh/ZrO_2_ > CoMoS_2_/Al_2_O_3_ > Pd/ZrO_2_ > Pt/ZrO_2_. Up to now, the basic mechanism of the transition metal in the HDO reaction is still unclear. It is accepted that the metal plays a role in hydrogen donation, but no conclusion has been established as to the activation mechanism for oxygen compounds.

### 5.4. Phosphide, Carbide, and Nitride Catalysts

Phosphide catalysts have garnered significant attention for their potential in the hydrodeoxygenation (HDO) of bio-oil, mainly due to their efficiency in oil hydrotreating, characterized by low activation energies and high catalytic activity [36,64,75,82,83,84]. A study by Gonçalves et al. [85] compared the effects of silica (SiO_2_) and tetragonal zirconia (ZrO_2_) as supports for nickel metal and nickel phosphide (Ni(0) and Ni_2_P) catalysts on the HDO of *m*-cresol at 340 °C and 4 MPa. The results showed that Ni_2_P phase was considerably more active than metallic Ni, and that zirconia supports improved the deoxygenation properties more effectively than silica supports, probably due to the oxophilic sites of zirconia (Zr^3+^/Zr^4+)^ that enhance the adsorption of *m*-cresol.

Additional insights were provided by Berenguer et al. [86] that studied the catalytic HDO of *m*-cresol using Ni_2_P supported on hierarchical zeolite (h-ZSM-5) at 200 °C and 25 bar H_2_. This setup induced the formation of strong Lewis acid sites, proportional to the Ni_2_P charge, complemented by new Brönsted acid sites due to P-OH units [82,83,87]. The Ni_2_P/h-ZSM-5 catalyst showed high selectivity (>97%) for converting *m*-cresol to methylcyclohexane, significantly improving compared to the Ni_2_P/SiO_2_ reference catalyst. This demonstrates the synergistic effects between the metal phosphide and the solid acid support, with catalyst activity displaying a dependence on Ni_2_P dispersion and optimal activity observed for particle sizes of 4 nm.

In parallel, alternative catalysts, such as carbides and nitrides, are being explored for their cost-efficiency and properties similar to traditional HDO catalysts. The research by Boullosa-Eiras et al. [88] investigated the performance of several catalysts, including carbide, nitride, phosphide and molybdenum oxide supported on titanium, in the HDO of phenol at 350 °C and 25 bar H_2_. Molybdenum carbide on titanium dioxide (Mo_2_C/TiO_2_) showed the highest activity with marked selectivity towards benzene. Moreover, molybdenum phosphide on titanium dioxide (MoP/TiO_2_) proved propensity to hydrogenate the aromatic ring and exhibited considerable selectivity towards methylcyclopentane, suggesting the influence of the acidic surface chemistry on the reaction behavior.

Although phosphide, nitride, and carbide catalysts demonstrate efficient performance in bio-oil HDO [88,89,90], their industrial scale adoption is still in the early stages, and they do not yet match the performance of commercial sulfide catalysts in industrial applications. Further research and development are needed to optimize these new catalysts for wider industrial deployment.

### 5.5. Ni_2_P Promoted Catalysts

A new class of hydrotreating catalysts known as transition metal phosphides has been developed and widely applied in processes like hydrodesulfurization (HDS) and hydrodenitrogenation (HDN), which are closely related to hydrodeoxygenation (HDO) [63,91]. Among them, nickel phosphide (Ni_2_P) has emerged as a particularly effective material due to its unique structural and chemical properties. Ni_2_P is characterized by a high density of Ni-Ni and Ni-P bonds, which contribute to its superior electronic conductivity, thermal stability, and chemical durability [82]. In addition, Ni_2_P displays a reduced propensity for hydrogenolysis of C–C bonds, a typically undesirable reaction that leads to the formation of short-chain hydrocarbons with low octane values [83].

The effectiveness of Ni_2_P reaches out to its role in facilitating the easy dissociation of hydrogen due to the electronic (ligand) and geometrical (assembly) effects imparted by the presence of phosphorus. This feature significantly enhances the hydrogenation activity of Ni species, making Ni_2_P a useful catalyst in the HDO process [87]. Recent studies focusing on the HDO of lignin-derived bio-oil model compounds have shown that Ni_2_P is a bifunctional catalyst system. In this system, Ni metal sites actively promote the hydrogenation of aromatic rings and the hydrogenolysis of C-O bonds, depending on the reaction conditions. At the same time, the phosphorus species of the catalyst generate Brønsted acid sites through the formation of P-OH groups, thus facilitating the direct HDO of methoxy or hydroxyl groups [85,92].

**Figure 8:**
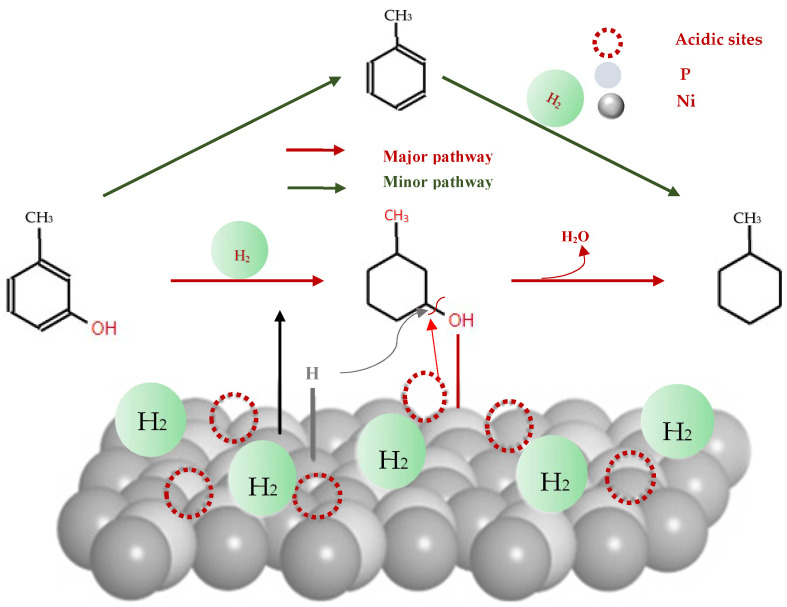
Catalytic hydrodeoxygenation performance of m-cresol over an Ni_2_P/SiO_2_ catalyst, adapted from [83].

Additional comparative analyses have underscored the higher activity of Ni_2_P-based catalysts compared to other transition metal phosphide catalysts in guaiacol HDO. Also, compared to Ni-based monometallic catalysts, Ni_2_P-based catalysts have consistently demonstrated superior catalytic performance [87]. Zhu et al. [83] specifically examined the impact of Ni and Ni_2_P active phases on the HDO of *m*-cresol at 275 °C for 120 min in a packed bed reactor under a hydrogen pressure of 3.0 MPa. The results of this study revealed that the Ni_2_P-based catalysts exhibited high and stable activity, reaching *m*-cresol conversion rates of over 94.7% at temperatures between 250 and 275 °C (Figure 8).

These results confirm the pivotal role of Ni_2_P in enhancing the field of catalytic HDO, emphasizing its potential to outperform traditional monometallic catalysts and other transition metal phosphide catalysts in complex biochemical conversions. Moreover, the catalytic performance of Ni_2_P-based catalysts for HDO of model compounds such as guaiacol [87], phenol [93] anisole [84], cresol [86,88], and real pyrolysis oil [62] has been extensively explored (Table 4).

Studies investigating the catalytic activity of Ni_2_P-based catalysts have demonstrated their remarkable selectivity towards saturated hydrocarbons suitable for use as transport molecules. For instance, Wang et al. [94] explored the HDO of furfural at 240 °C and 2.0 MPa for 4 h using MoP or Ni_2_P catalysts. They found that using Ni_2_P_0.5% as catalyst resulted in a high selectivity of 83.1% towards 2-methylfuran, together with a complete conversion of furfural. Similarly, Berenguer et al. [86] carried out the HDO of phenol in a batch reactor at 200 °C, 4 MPa and 2 h reaction time using Ni_2_P supported on Al-SBA-15. Their results showed a complete conversion of phenol (100%) and a high selectivity of 91% towards cyclohexane.

**Figure 9:**
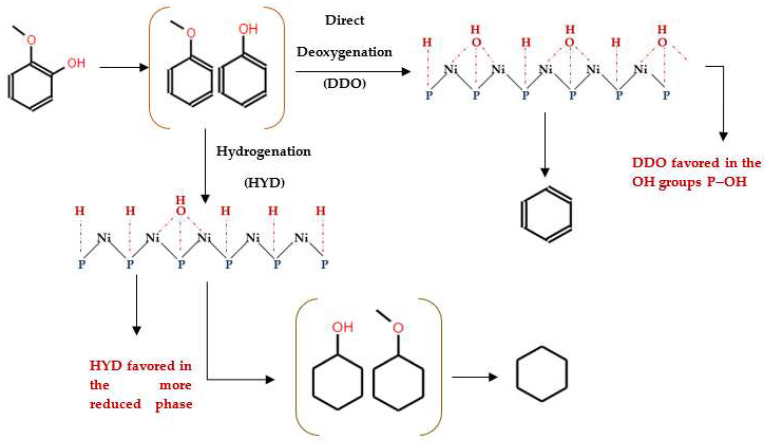
Proposed reaction pathway for HDO of guaiacol to hydrocarbons over Ni_2_P-based catalyst, adapted from [63,87].

Moreover, Moon et al. [87] proposed a mechanism for the conversion of guaiacol to cyclohexane and benzene (Figure 9). According to their model, the conversion of guaiacol over Ni_2_P-based catalysts initiated with direct deoxygenation, which produced anisole and phenol as primary intermediate species. At a low pressure, direct deoxygenation (DDO) of these intermediate species prevailed, leading to the formation of benzene. However, at a high hydrogen pressure, hydrogenation of the aromatic ring became dominant, leading to the formation of cyclohexanol and methoxycyclohexane. Subsequent deoxygenation processes resulted in cyclohexane as the final hydrocarbon product observed over the Ni_2_P catalyst.

Interestingly, both reaction pathways underscore the dual catalytic functions of Ni_2_P-based catalysts. The Brønsted acid sites formed from surface hydroxyl groups (OH) facilitate deoxygenation reactions (C–O bond cleavage) (Figure 9), while the Ni redox sites enhance hydrogenation processes [87,94]. Additionally, Ni_2_P is characterized by abundant Ni–Ni and Ni–P bonds, which contribute to its superior electron conductivity, as well as its thermal and chemical stability [82]. Notably, Ni_2_P has a lower propensity for hydrogenolysis of C–C bonds, minimizing the production of low-octane short-chain hydrocarbons, an undesirable side reaction [83]. The presence of phosphorus in the structure induces both electronic (ligand) and geometric (assembly) effects on Ni sites, enabling efficient hydrogen dissociation and thus boosting the hydrogenation activity of Ni species [87]. This mechanistic insight is crucial for understanding the catalytic properties of Ni_2_P-based catalysts and their potential use in converting lignin-derived bio-oils into valuable transportation fuels.

The activity of Ni_2_P catalyst was also investigated in a series of experiments for guaiacol HDO compared to a wide variety of other metal phosphides including Co_2_P, Fe_2_P, WP, and MoP. An order of turnover frequency of active sites, Ni_2_P > Co_2_P > Fe_2_P, WP, and MoP, was observed by CO titrating chemisorption [95]. Table 4 presents the HDO of various feedstocks over Ni and Ni-modified supported catalyst.

**Table 4:** Summary of HDO results of bio-oil and model compound using Ni and Ni-modified supported catalyst.

Catalysts	Feedstock	T (^o^C)	P (MPa)	T (h)	Setup	Conversions mol. %	Major Products	Selectivitymol. %	Refs.
Ni_2_P/SiO_2_	*M*-cresol	250	3	1	Batch	94.7	Hydrocarbons	~96.3	[83]
Ni_2_P/Zr-SBA-15	Bio Oil	330	4,5	4	Fixed-bed	98	Hydrocarbons	67.80	[96]
Ni_2_P/Fe-SBA-15	Benzofuran	300	3.0	7	Fixed-bed	91.7	Hydrocarbons	83.3	[97]
Ni_2_P/SiO_2_	Furfural	200	0.1	2	Fixed-bed	90	Hydrocarbons	~60	[98]
Ni_2_P/2D ZSM-5	Guaiacol	260	4	2	Batch	78	Cyclohexane	95.0	[99]
Ni_2_P/Al_2_O_3_-ZSM-5	Methyl palmitate	340	2	-	Continuous reactor	80.3	Isoalkanes (i-C_15_-i-C_16_)	62.1	[100]
Ni_2_P/AC	Waste cooking oil	300	0.1	~1	Continuous reactor	85	Hydrocarbons(n-alkanes)	~60	[101]
Ni_2_P/MCM-41	γ-Valerolactone	350	0.5	3	Continuous reactor	~100.0	Hydrocarbons (Butane)	88.0	[102]
Ni_2_P@C(x)	Phenol	250	2	2	Batch	100	Cyclohexane	100	[103]
PdNi_2_P/SiO_2_	Phenol	220	2	3	Fixed-bed	100	Cyclohexane	98	[104]
Ni_2_P/HZSM-5	*M*-cresol	200	2.5	6	Batch	97	Methylcyclohexane	88	[86]
Ni_2_P/HZS M-5	4-ethylguaiacol	400	0.5	8	Continuous flow reactor	84	Hydrocarbons	65.10	[25]
Ni/HZSM-5&La	Guaiacol	350	2	0.83	Fixed-bed	97.79	Hydrocarbons	61.75	[105]
Ni_2_P/H-ZSM-5	Oleic acid	300	5	6	Batch	65	Hydrocarbons	29	[106]
NiP(2:1)/NZ0.5	PFAD	350	4	2	Fixed-bed	100	Hydrocarbons	93.32	[107]
Ni_2_P/USYZ	Oleic Acid	340	1	1	Batch		Hydrocarbons	48	[108]
Ni_2_P/ZSM-5	Blends	260	0.4	-	Batch		Cyclohexane & Ethane		[109]
Ni_2_P/HZSM-22	Palmitic acid	350	0.1	2.5	Fixed-bed	99.6	Hydrocarbons	42.9	[110]
Ni_2_P/HZSM	Bio oil	450	0.5	1.30	Fixed-bed	80	Hydrocarbons	28.87	[25]
In-situ Ni_2_P	Terephthalic acid	400	7	6	Autoclave	98	Benzene-toluene-xylene	100	[111]
Ni_2_P@C-T	Furfural	150	1	4	Batch	100	N-butyl furfufurylamine	85	[112]
Ni_2_P@C/FLRC-TiO_2_	*p*-cresol	275	2	1.5	Batch	100	4-methylcyclohexanol	90.8	[113]

## 6. Hydrodeoxygenation of Model Compounds

The complexity of bio-oil composition poses significant challenges in understanding its HDO pathways, due to the simultaneous occurrence of numerous reactions during the upgrading process. Consequently, many researchers have chosen to use model compounds instead of full-scale pyrolysis oil in their studies. These model compounds cover a wide range of classifications, including phenols such as guaiacol [87], phenol [93], and cresol [86], as well as aldehydes such as furfural [114], ethers such as anisole [115,116], furans such as 2-methylfuran [117], carboxylic acids such as acetic acid [109], and ketones such as acetone [118], among others. For example, Zhu et al. [83] investigated the HDO of *m*-cresol using Ni-based catalysts in a fixed-bed reactor, achieving a complete conversion to methylcyclohexane (MCH) at 250 °C. These experiments with model compounds are essential for gaining comprehensive insights into HDO reaction mechanisms and networks, as well as for the selection and design of efficient catalysts [83].

**Figure 10:**
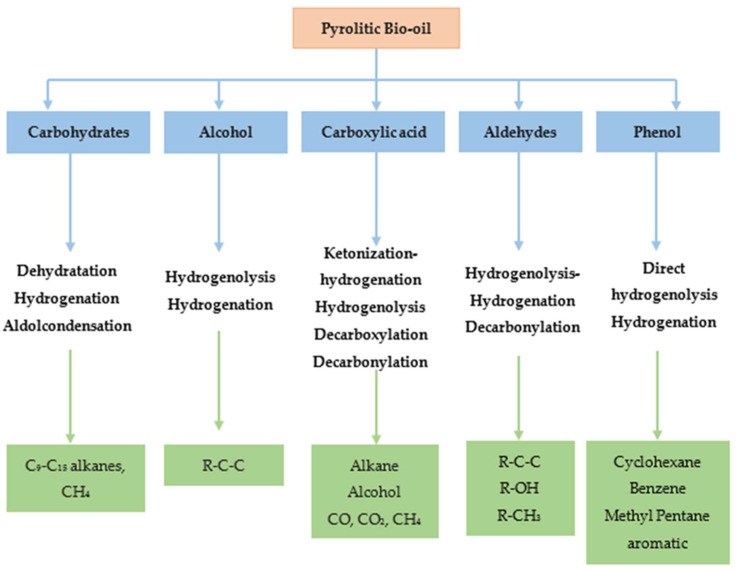
Possible reactions in the HDO of model molecules, adapted from [119].

At present, model compounds are meticulously chosen to represent the most active components of bio-oil, a factor contributing to its inherent instability. These compounds, characterized by a wide range of functional groups, facilitate the exploration of relative activities and selectivity in various reactions, encompassing dehydration, decarboxylation, hydrogenation, hydrogenolysis and hydrocracking (see Figure 6). Additionally, dimeric compounds are selected as model compounds to provide valuable information about the cleavage of major linkage types prevalent in bio-oil. Through the utilization of these model compounds, researchers can further investigate the fundamental reactions underlying HDO, as depicted in Figure 10, and design strategies to optimize the performance of catalysts for the efficient conversion of bio-oil into valuable fuels and chemicals.

### 6.1. Hydrodeoxygenation of Phenols and Alkylated Phenols (Guaiacols)

Phenolic monomers, such as phenols, guaiacols, and syringols, represent fundamental by-products resulting from lignin degradation. Among these, phenol and its alkylated derivatives (cresol and 2-ethylphenol) stand out as the most prevalent lignin-derived phenolic monomers. The HDO process, essential for converting lignin-derived phenols, primarily involves the cleavage of the C-OH bond. This process can follow two different chemical routes, leading to the production of cycloalkanes and arenes: (i) hydrogenation of the aromatic ring followed by deoxygenation of the alcohols to produce cycloalkanes, and (ii) direct deoxygenation to generate arenes by cleaving the C-OH bond [93]. Hydrogenation, dehydration, and hydrogenolysis reactions represent the three main categories of reactions involved in the HDO of phenolic compounds [103].

**Figure 11:**
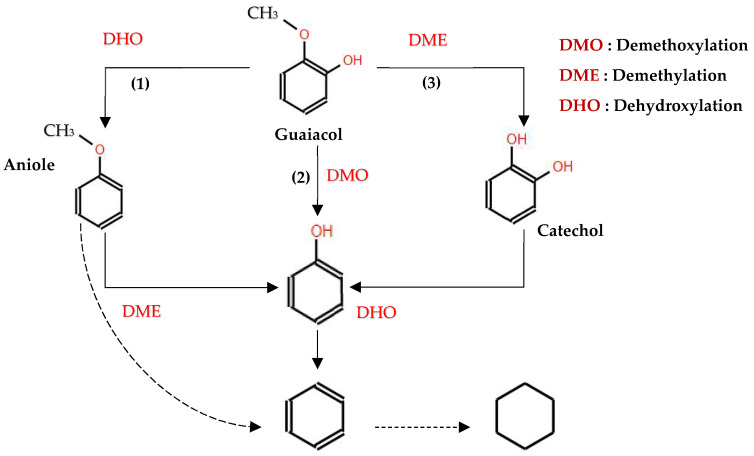
Reaction pathways and selectivity of aromatic products in the HDO of Guaiacol over Ni_2_P/SiO_2_ at 300 °C, adapted from [120].

In studies focusing on the HDO of phenols, Lan et al. [120] investigated the HDO of guaiacol, revealing that Ni_2_P/SiO_2_ exhibited a higher selectivity towards benzene. This selectivity was achieved through demethoxylation and dehydroxylation via phenol and anisole intermediates (over 97%), compared to the MoP/SiO_2_ catalyst. They also observed a significant influence Ni_2_P particle size on the turnover frequencies of guaiacol HDO, as illustrated in their proposed reaction pathways depicted in Figure 11. Similar results were reported from the hydrodeoxygenation of phenol in the liquid phase over a Rh/silica catalyst [121]. Additionally, Gutiérrez-Rubio et al. [99] explored the HDO of bio-derived anisole using different catalysts, including nickel supported on 2D zeolites (L-ZSM-5 and PI-ZSM-5), achieving high guaiacol conversion rates of 75% and 78%, respectively.

Complementary research by Kirkwood al. [122] examined the competitive hydrogenation and hydrodeoxygenation (HDO) of dihydroxybenzene isomers, catechol (1,2-dihydroxybenzene), resorcinol (1,3-dihydroxybenzene), and hydroquinone (1,4-dihydroxybenzene) over a Rh/silica catalyst. Their findings indicated that the use of deuterium had a significant effect on HDO energetics, with reactions significantly inhibited, meaning that hydrogenation and HDO are mechanistically separate. This effect on reaction energetics achieved when more than one substrate was involved underscores the limitations of studying a single model compound as a way to understand the processes necessary for the upgrading of a real bio-oil.

### 6.2. Hydrodeoxygenation of Ketones, Aldehydes, and Alcohols

**Figure 12:**
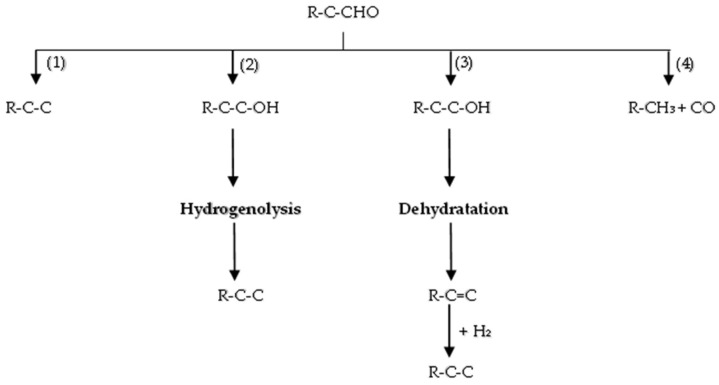
Main reaction pathways of HDO of aldehydes and alcohols; R stands for alkyl groups, adapted from [124].

Pyrolysis oil is composed of a diverse array of components, including ketones10–15%, carboxylic acids, phenols, guaiacols, and aldehydes 0–5% [15]. Among the oxygenated compounds found in pyrolysis oil, furan, tetrahydrofuran (THF), furanone, furfural, furfuryl alcohol, and 5-hydroxymethyl furfural (HMF) present notable challenges in terms of deoxygenation. The HDO of aldehydes, such as furfural, can theoretically progress through four major routes [123], as depicted in Figure 12 and enumerated as following:

Direct hydrogenolysis of the C=O bond [98];

Hydrogenation of C=O bond to form alcohols, followed by hydrogenolysis of the C-O bond to produce alkanes (hydrogenation-hydrogenolytic mechanism);

Hydrogenation of C=O bond to form alcohols, subsequent dehydration to generate olefins, and then rehydrogenation of the C=C bond to yield corresponding alkanes (hydrogenation-hydrogenolytic mechanism);

Decarbonylation of C=O to generate CO and alkane with one fewer carbon,

These routes involve the conversion of the carbonyl group into a methyl group or CO. The conversion of alcohols encompasses steps 2 and 3, and ketone conversion entails all steps but is not depicted in Figure 12 due to differing molecular formulas. These mechanistic insights serve as a basis for understanding the intricate transformations involved in the HDO of aldehydes, highlighting both the challenges and opportunities in developing efficient catalysts for the valorization of pyrolysis oil constituents.

**Figure 13:**
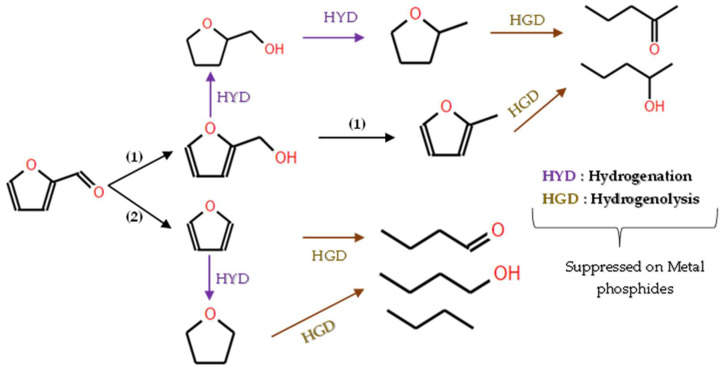
Reaction pathways in the HDO of furfural according to [114]. FOL: furfuryl alcohol, MF: 2-methylfuran, THF: tetrahydrofuran, THMF: tetrahydro-2-methylfuran, THFOL: tetrahydrofurfuryl alcohol, adapted from [98].

In the realm of hydrodeoxygenation (HDO), various studies have investigated the conversion of oxygenated organic compounds into valuable chemicals and fuels. For instance, Lan et al. [98] conducted a study on the HDO of furfural using bifunctional Ni_2_P catalysts in a flow fixed-bed reactor at 1 bar pressure and 200 °C and a weight hourly space velocity (WHSV) of 3 h^−1^, and they achieved a 90% conversion rate of furfural and identfied a highly selective pathway leading to 2-methylfuran (MF), as illustrated in Figure 13.

Complementary research by Wang et al. [125] explored the HDO of furfural over a modified Cu/ZSM-5 catalyst incorporated with nickel (Ni). Their findings revealed that nickel’s moderate addition enhanced both the adsorption of furfural and hydrogen, thereby accelerating the conversion process. This modification resulted in a significant increase in selectivity for 2-Methylfuran (2-MeF), reaching 78.8 wt% at 220 °C after 30 min of reaction. The reaction pathway involved a sequence of hydrogenation followed by deoxygenation steps.

Simultaneously, Lino et al. [126] investigated the HDO of 2-methyltetrahydrofuran using Ni_2_P supported on SiO_2_ in a continuous-flow fixed bed quartz reactor at 0.5 MPa of N_2_ and high nickel loading, which were conducive to furfural conversion at elevated temperatures. A notable finding was that a higher partial pressure of H2 markedly favored the hydrogenation process, leading to a total furan yield surpassing 100% and a selectivity of 85% to n-pentane at 350 °C.

**Figure 14:**
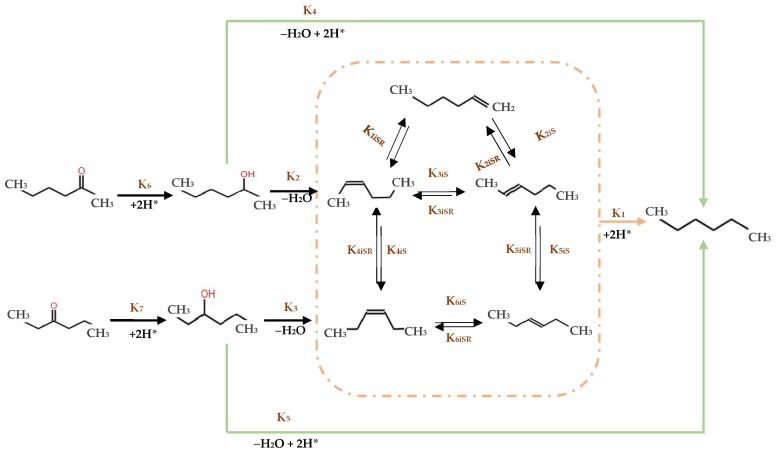
Proposed reaction scheme of the HDO of model compounds: 2-hexanone, 2-hexanol, 3-hexanone, and 3-hexanol over a NiMo/Al_2_O_3_ catalyst, adapted from [127].

Further explorations in HDO were conducted by researchers using NiMo/γ-Al_2_O_3_ catalysts supported on sulfides in a batch reactor [127] at 5 MPa H2 and 250 °C, primary transformations of 2-hexanone and 3-hexanone involved hydrogenation into their respective alcohols, followed by dehydration to form olefins. These were subsequently hydrogenated into saturated alkanes, predominantly hexane. An alternative pathway featured the direct hydrogenolysis of the C-O bond, crucial for the conversion of secondary hexanol into hexane at 275 °C (Figure 14). Notably, while the presence of various hexene isomers was consistent with literature finding [128], only trace amounts of 1-hexene were detected, suggesting its formation through isomerization of 2-hexene. Ultimately, this sequence culminated in the hydrogenation of all hexene isomers into hexane, delineating the primary product pathway from alcohols and ketones [127,128].

These studies collectively underscore the influence of catalyst composition, reactor conditions, and hydrogen pressure in optimizing HDO processes, each contributing uniquely to the enhanced conversion rates, selectivity, and understanding of reaction mechanisms in converting ketones, aldehydes, and alcohols compounds into hydrocarbons.

### 6.3. Hydrodeoxygenation of Carboxylic Acids

**Figure 15:**
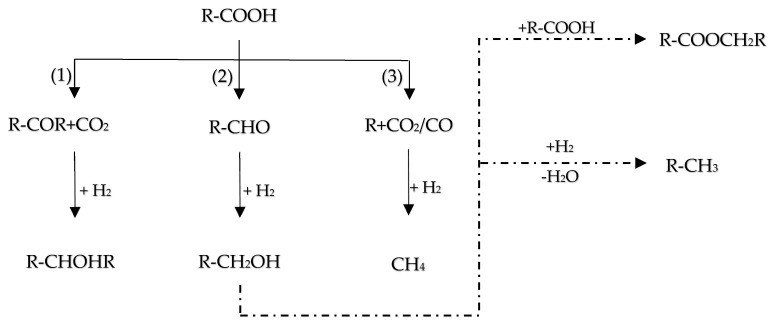
General main reaction pathways of HDO of carboxylic acids; R stands for alkyl groups, adapted from [124].

The inherent acidity of bio-oils, often manifested by a pH between 2 and 3 [129], primarily arises from the presence of carboxylic acids such as acetic and formic acids. This acidic nature significantly contributes to the corrosive properties of bio-oils, particularly under elevated temperatures, as noted in [109]. In the HDO of these carboxylic acids, the literature has described three principal reaction pathways (as delineated in Figure 15):

(1) Ketonization by C-O bond cleavage to generate ketones, and further by hydrogenation to produce alcohols.
(2) Hydrogenolysis by the C-O bond cleavage to yield aldehyde, followed by further hydrogenation to produce alcohols, and then dehydration and hydrogenation to obtain alkane, or the alcohols react with carboxylic acids to form esters.
(3) Decomposition (decarboxylation and decarbonylation) of carboxylic acids by breaking C–C bond to yield alkanes with one less carbon, CO and CO_2_. Also, CO can be further hydrogenated to methane.

A detailed investigation into the HDO of acetic acid using reduced sulfided Ni-Mo(R)/ZSM-5 catalysts [130] showed that HDO of palmitic acid could occur effectively below 300 °C with an initial hydrogen pressure of 35 bar. After 4 h, the conversion of palmitic acid neared 99%, producing a range of isomerized paraffins with carbon numbers from 6 to 16, as identified by GC-MS. This observation suggests that the catalyst facilitates both hydrodeoxygenation and hydroisomerization reactions concurrently on palmitic acid.

**Figure 16:**
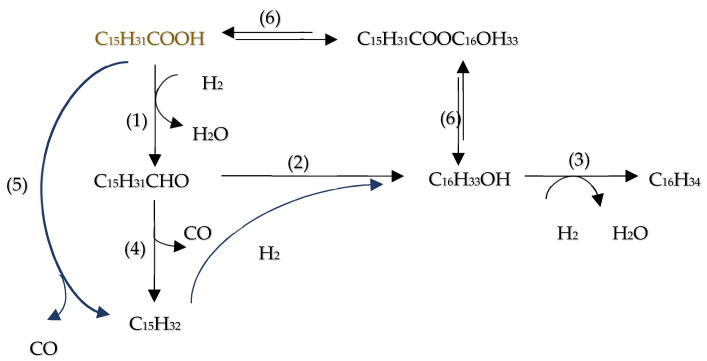
Proposed reaction network over Ni_2_P/Al_2_O_3_ catalyst; the reaction steps are (1) Hydrogenolysis, (2) Hydrogenation, (3) Dehydration–hydrogenation, (4) Decarbonylation, (5) Decarboxylation, (6) Esterification, adapted from [131].

Furthermore, Peroni et al. [131] examined the HDO of formic acid using a temperature-programmed reaction (TPR) approach with a Ni_2_P/Al_2_O_3_ catalyst in a bed flow reactor under various temperatures and residence times. Their study demonstrated a complete conversion of palmitic acid, where the use of Ni_2_P supported on Al_2_O_3_ increased the selectivity towards decarbonylation and decarboxylation processes. Remarkably, this resulted in pentadecane being the predominant product, constituting about 78% of yield. According to the hydrodeoxygenation mechanism presented in Figure 16, initial products included pentadecane and hexadecane, which subsequently underwent decarbonylation to yield n-pentadecane and CO according to [132].

These studies collectively illuminate the nuanced dynamics of HDO mechanisms under varying conditions and catalyst formulations, elucidating how such parameters dictate the efficiency, selectivity, and range of products formed during the deoxygenation of carboxylic acids in bio-oils.

### 6.4. Hydrodeoxygenation of Carbohydrates

**Figure 17:**
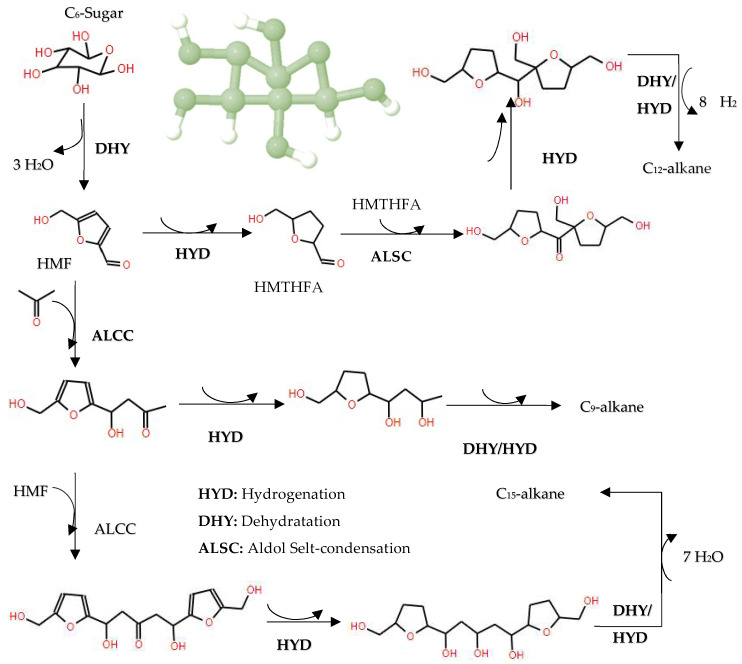
Reaction pathways for the conversion of carbohydrates biomass-derived glucose into liquid alkanes, adapted from [133].

In the domain of hydrodeoxygenation of carbohydrates, a detailed understanding of the reaction pathways and catalyst functionalities is critical for optimizing the production of biofuels and chemicals. Initially, the HDO of carbohydrates such as C6 sugars (e.g., fructose) typically begins with dehydration to form 5-hydroxymethylfurfural (HMF), a pivotal intermediate in the conversion process. Subsequently, HMF undergoes hydrogenation to yield 5-(hydroxymethyl)tetrahydrofuran-2-carbaldehyde (HMTHFA), as delineated in [133]. These intermediates can participate in further reactions such as aldol condensation, leading to the formation of larger molecules, and are subject to multistep hydrogenation and dehydration processes to synthesize C9–C15 alkanes, as illustrated in Figure 17.

**Figure 18:**
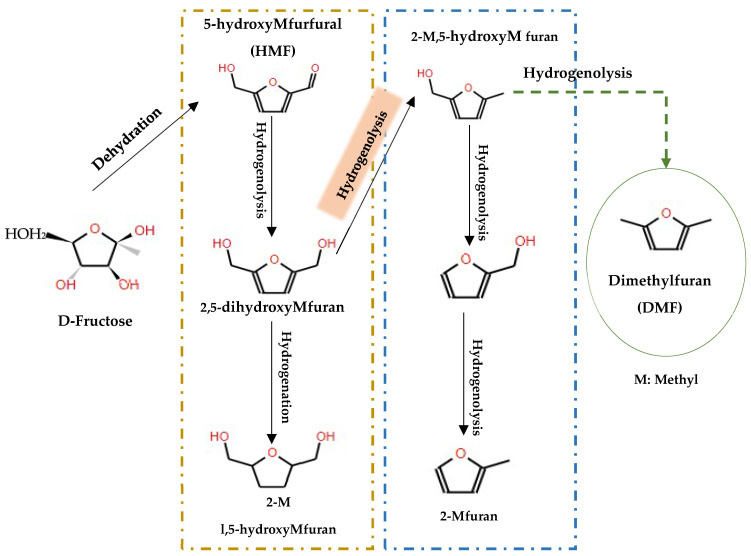
Reaction pathways for the conversion of carbohydrates) formed by removal of oxygen atoms from hexoses (fructose), adapted from [134].

A variety of catalysts with metal and acid sites, such as CuRu/C, have been used for the initial dehydration of fructose (C6-sugar) to HMF, followed by hydrogenolysis of the C-O bonds inside HMF to produce 2,5-dimethylfuran (DMF) [134], as illustrated in Figure 18. This step is crucial as DMF serves as a gateway for other hydrogenated products. In addition, research involving different metal sites supported on silica, including Ni, Cu, Fe, Co, Pt, Pd and Re, has demonstrated that all these metals are capable of producing DMF from HMF using 1-propanol as solvent in a tubular flow reactor at 180 °C and 33 bar, according to the results of [134,135]. Moreover, the transformation of DMF can yield a variety of products depending on the metal employed; for example, ring-opening and 2,5-dimethyltetrahydrofuran (DMTHF) products have been specifically observed with Pd/C catalysts.

Parallel to these developments, other catalyst such as acid catalysts (Zr-P, SiO_2_-Al_2_O_3_, WO_x_/ZrO_2_, γ-Al2O_3_, and HY zeolite) have also been used for aqueous-phase HDO of carbohydrate (xylose) at 160 °C in a batch reactor [136]. The dehydration of xylose predominantly leads to the production of furfural. The efficiency of this conversion, particularly the selectivity towards furfural after 30 min, is significantly; about 20% influenced by the ratio of Brønsted to Lewis acid sites on the catalyst. Notably, catalysts like Zr-P exhibit a furfural selectivity substantially higher than those with predominately Lewis acid sites, even at modest xylose conversion levels. This high selectivity is comparable to that achieved with ion-exchange polymer resins featuring high concentrations of Brønsted acid sites, similar to Zr-P and HCl. The presence of Lewis acid sites, however, tends to catalyze side reactions between xylose and furfural, leading to the formation of undesired by-products such as humins.

These studies collectively emphasize the importance of catalyst composition—balancing metal and acid sites—and reaction conditions in steering the HDO pathways from simple sugars to complex hydrocarbons and chemicals. Understanding these dynamics facilitates the development of tailored catalyst systems that enhance selectivity and yield, crucial for the efficient conversion of biomass into valuable biofuels and chemical feedstocks.

## 7. Hydrodeoxygenation of Mixtures

**Figure 19:**
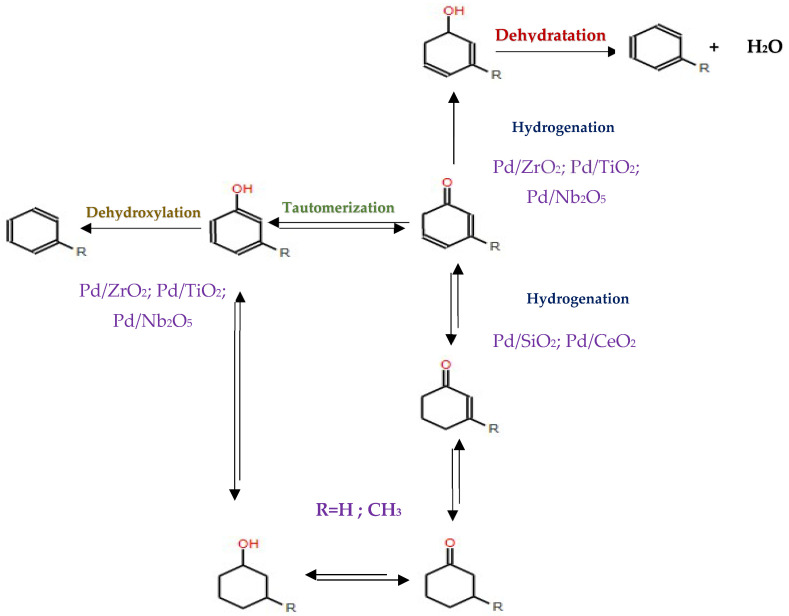
Reaction scheme for the hydrodeoxygenation of phenolic compounds (phenol and m-cresol) for the Pd catalysts supported on various oxides, adapted from [137].

Research into the hydrodeoxygenation of binary mixtures containing aromatic compounds has shown intriguing results concerning the interaction and competitive adsorption behaviors on catalyst surfaces. Teles et al. [137] conducted studies on binary mixtures (phenol/anisole and *m*-cresol/anisole) using Pd catalysts supported on various oxides like SiO_2_, CeO_2_, ZrO_2_, and TiO_2_. Their findings indicated that the hydroxyl (OH) groups in phenol and *m*-cresol exhibited stronger adsorption to the catalyst surfaces compared to the methoxy groups of anisole. This preferential adsorption facilitated the reactions of the hydroxyl-containing molecules. Despite the apparent competition for active sites on the catalyst, the interaction between the model compounds was characterized as weak, as depicted in Figure 19. Similarly, Funkenbusch et al. [138] observed comparable behavior in the HDO of other blends (anisole/*m*-cresol and anisole/phenol) over Pt/Al_2_O_3_ and Pd/C catalysts, reinforcing the concept that the nature of functional groups significantly influences adsorption and subsequent reactivity in these systems.

Extending the complexity of the reagent mixtures, Roldugina et al. [139] explored the HDO of a synthetic bio-oil mixture containing guaiacol, water, dodecane and methanol, using a ruthenium catalyst supported on aluminum-modified hexagonal mesoporous silica (Ru/Al-HMS), performed under a hydrogen pressure of 6.0 MPa and a temperature of 250 °C. The upgraded bio-oil exhibited a conversion rate of 38% at 210 °C, yielding major products such as cyclohexanol (47%), phenol (18%), benzene (15%), and cyclohexane (7%). However, when the temperature was increased to 290 °C and the hydrogen pressure was reduced to 2.5 MPa, the conversion of guaiacol dropped to 19%, with phenol being the predominant product with a selectivity of 70%.

These studies collectively demonstrate the critical roles of catalyst composition, reactant structure, and reaction conditions in dictating the dynamics of HDO processes. The selective adsorption of functional groups and the subsequent reactivity highlight the nuanced interactions within the catalytic environment, essential for optimizing conversion and selectivity in the transformation of complex bio-oil mixtures to valuable chemical products.

### Hydrodeoxygenation of Mixtures over Zeolites and Non-Noble Metal Catalysts

**Figure 20:**
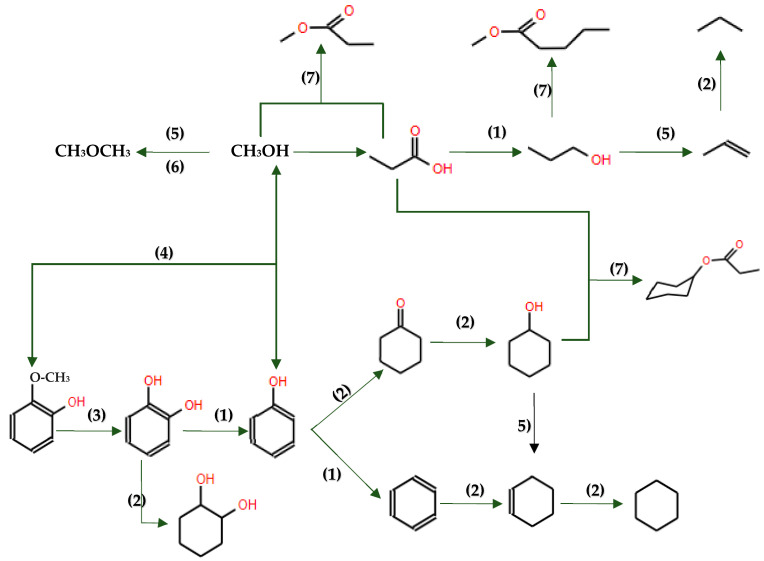
Product distribution and major pathways of guaiacol and propionic acid transformations in HDO conditions: (1) Hydrogenolysis, (2) Hydrogenation, (3) Demethylation, (4) Demethoxylation, (5) Dehydration, (6) Etherification, (7) Esterification, adapted from [140].

Investigations on the hydrodeoxygenation (HDO) of bio-oil mixtures have revealed the effectiveness of non-noble metal catalysts supported on solids with varying acidity and textural properties due to their high HDO activity and reduced cost. Sankaranarayanan et al. [140] addressed on the catalytic transformation of mixtures containing guaiacol and propionic acid over Ni-based catalysts supported on solids such as hierarchical ZSM-5 (h-ZSM-5), SBA-15, and Al-SBA-15. These catalysts assisted the in situ formation of methanol from guaiacol demethoxylation and the generation of other alcohols such as cyclohexanol, enabling the esterification of carboxylic acids without external alcohol addiction. This approach allowed for significant upgrading of carboxylic acids directly within the HDO process, demonstrating the high efficacy of Ni/h-ZSM-5 in both HDO and esterification, with methyl propionate emerging as a major product. Methyl propionate has been identified as the major product of propionic acid transformation over all catalysts (Figure 20).

**Figure 21:**
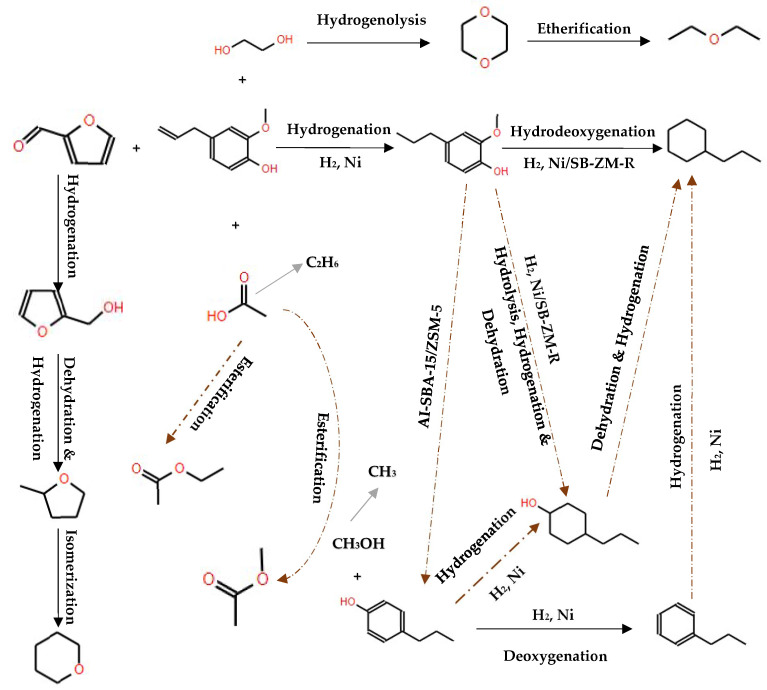
Major reaction pathways of eugenol and acetic acid, ethylene glycol, and furfural transformations during the HDO process, adapted from [141].

Further exploring the role of Ni-based catalysts, Chen et al. [141] examined the impact of catalyst composition on the HDO of a mixture of eugenol with light bio-oil fractions using Ni/SB-ZM-R, Ni/SB-R, and Ni/ZM-R. They observed that the addition of different molecular fractions significantly affected the selectivity of products like propylcyclohexane. Among the catalysts tested, Ni/SB-ZM-R showed superior performance due to its optimal pore structure and acidity. The presence of other compounds, like ethylene glycol, increased the selectivity for propylcyclohexane from 67.9% up to 90%, whereas additives like furfural and acetic acid (1%) significantly decreased its selectivity to 36%, indicating their adverse effects on HDO efficiency. The study delineated two different reaction pathways that could be seen for the eugenol: (i) eugenol was converted into 2-methoxy-4-propylphenol and then converted into the final product propylcyclohexane via HDO, and (ii) 2-methoxy-4-propylphenol was firstly converted into 4-propenebenzene and then converted into propylcyclohexane by hydrogenation. For the other pathway, 4-propylcyclohexanol was obtained by hydrolysis, dehydration, and hydrodehydration, leading to final products such as propylcyclohexane (Figure 21).

In another investigation focusing on a mixture of guaiacol and acetic acid, the researchers used a Ni_2_P/ZSM-5 catalyst [109]. This study revealed a partial HDO inhibition of guaiacol due to competitive adsorption of acetic acid on the active sites of the catalyst. Ni_2_P incorporation altered the acidic properties of ZSM-5 by introducing new acid sites of moderate strength, consisting of Ni^δ+^ species (Lewis acid sites) and residual P-OH groups (weak Brønsted acid sites). This modification affected the adsorption capacity and performance of the catalyst in microporous and mesoporous structures differently [99]. However, this system also facilitated positive interactions such as esterification, leading to the production of guaiacol acetate, and acylation reactions producing acetophenones, especially apocynin [99].

Collectively, these studies illustrate the nuanced interplay between catalyst properties, reagent composition and reaction pathways in the HDO of complex bio-oil blends. They emphasize the importance of catalyst design and selection based on specific feedstock compositions and desired chemical performance, highlighting the potential of tailored catalyst systems to optimize both performance and selectivity in renewable energy applications.

## 8. Vegetal Bio-Oil Hydrodeoxygenation over Zeolites and Non-Noble Metal Catalysts

**Figure 22:**
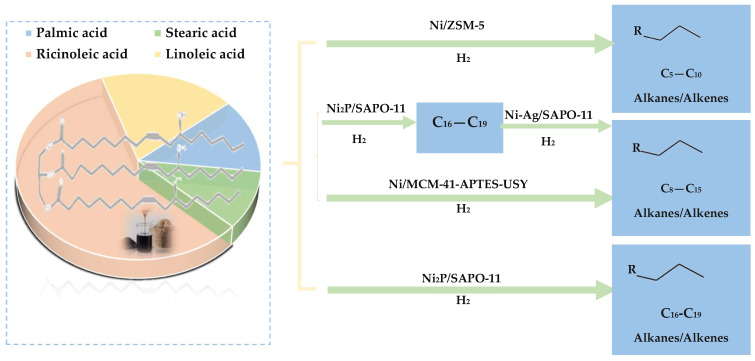
Hydroprocessing castor oil by Ni-based bifunctional catalysts with variable acidity, adapted from [145].

Hydrodeoxygenation from vegetable bio-oils, particularly using zeolites and non-noble metal catalysts, has demonstrated significant progress in improving the yield and quality of bio-derived fuels. For instance, Tang et al. [142] documented the HDO of jatropha oil using a 5 wt% Ni-Fe/SAPO-11 magnetic catalyst in a stainless-steel reactor, where they reported high HDO activity. The catalyst effectively produced various hydrocarbon types in the product oil, including linear alkanes (59.83%), isoparaffins (4.16%), aromatics (8.41%), and naphthenes (12.03%). In particular, the oil contained a high proportion of biofuel (C8–C16; 55.04%), and the overall deoxygenation rate achieved was 95.48% after 6 h of reaction (Figure 22).

Likewise, Wu et al. [143] examined the HDO of fatty acid methyl ester (FAME) over a Ni/SAPO-11-X catalyst. They obtained a high selectivity for C15–C18 products, reaching 94%. They achieved high selectivity for C15–C18 products, reaching 94%, with the oxygen content in the feed removed mainly in the form of CO_2_ and CO. In comparison to a lower performance using a Pt/Al_2_O_3_-SAPO-11 catalyst [144], indicating the performance of the Ni-based catalyst in promoting deoxygenation.

Additional investigations by Luo et al. [146] on the HDO of Jatropha curcas oil with Ni_2_P/MCM-41 and Ni_2_P/Zr-MCM-41 catalysts at 20 wt% in a trickling bed reactor resulted in high HDO activity. The significant conversion rate reached 93.90%, and the product oil was predominantly composed of linear paraffins (85.36%), among which the diesel fraction (C15–C20) exceeded 61.90%, especially when using the Ni_2_P/Zr-MCM-41 catalyst with a Ni_2_P loading of 20%.

Following these results, another study focused on the synthesis of aviation biofuel by HDO of Jatropha oil on nickel-based bimetallic catalysts in a fixed-bed reactor [147]. This process resulted in a high yield of aviation range alkanes (C6–C18) at 63.5 wt%, with smaller fractions of *i*-paraffins, aromatics, and naphthenes. The predominant products, C15–C18 alkanes, were formed through hydrodeoxygenation, decarbonylation, or decarboxylation reactions, while the *i*-paraffins and aromatics were products of isomerization and aromatization reactions.

These studies collectively demonstrate the promising potential of using specific zeolite supported non-noble and bimetallic catalysts for the efficient production of high-value deoxygenated biofuels. The selection of catalyst composition and operating conditions plays a crucial role in determining the yield, product distribution and overall efficiency of the HDO process, underlining the importance of developing tailor-made catalysts for the production of renewable fuels.

## 9. True Bio-Oil Hydrodeoxygenation over Zeolites and Non-Noble Metal Catalysts

The HDO of biomass-derived pyrolysis oils has attracted increasing interest from researchers aiming to improve the quality and utility of bio-oils. A remarkable investigation on the HDO of fast pyrolysis beech wood oil (FPBO) using a nickel-based catalyst revealed the propensity for higher gas production at elevated temperatures, showing remarkable yields of carbon dioxide (by decarboxylation) and methane (by cleavage of the C–C bond) [62]. Gas chromatographic analyses indicated the complete conversion of ketones, furfural, and aldehydes, while aromatic compounds remained stable. In particular, the study showed that bimetallic nickel-chromium catalysts altered the properties of the upgraded bio-oil more efficiently than monometallic nickel catalysts, achieving a yield improvement of up to 42%.

Following the topic of effective catalysts in HDO processes, Ismail et al. [148] reported the selective formation of fuel components such as BTX (benzene, xylene, toluene) using a Ni/Ni-H-Beta zeolite catalyst in a multistage catalytic pyrolysis reactor. The operational optimal conditions identified were 400 °C, an initial hydrogen pressure of 4 MPa, and a reaction time of 20 min, yielding a dominant product set of alkylated benzenes and BTX compounds in the range of C7–C9 hydrocarbons, constituting approximately 75% of the products. The resulting bio-oil showed a significant increase in the higher heating value (HHV), from about 20 MJ/kg in its original state to 41.5 MJ/kg after upgrading.

**Figure 23:**
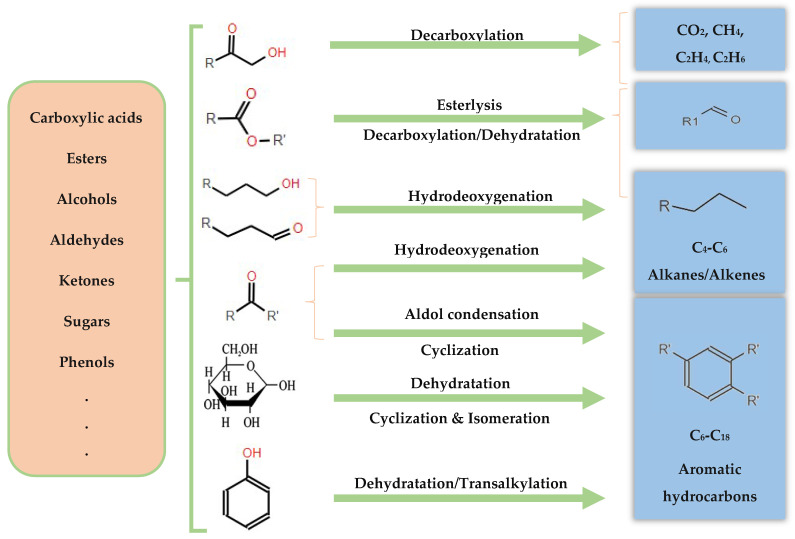
Main reaction pathways of bio-oil hydrotreatment over Ni_2_P/HZSM-5 catalysts, adapted from [64].

Further understanding of bio-oil upgrading, supplementary investigations with a Ni_2_P/HZSM-5 catalyst demonstrated effective deoxygenation pathways for crude bio-oil from beechwood pyrolysis [25]. The catalyst, with a Ni:P ratio of 1:2 and a Ni_2_P content of 5%, showed superior performance, resulting in the production of a bio-oil rich in aromatic hydrocarbons and phenolic compounds (Figure 23), suitable for use as a gasoline additive. This improved oil also showed a higher generation of gases such as CO, CH_4_, and CO_2_, similar results were obtained by other authors such as Wang et al. [64,103].

In another study, Shafaghat et al. [149] examined the HDO of crude bio-oil using supercritical fluids (ethanol, methanol, 2-propanol) in a high-pressure batch reactor equipped with a nickel catalyst supported on HBeta zeolite (10 wt.%). The use of supercritical methanol was especially effective, increasing the calorific value of the crude bio-oil from 12.61 MJ/kg to 24.17 MJ/kg. The maximum deoxygenation attained under these conditions was 46.37%, with an HHV reaching 26.04 MJ/kg at a hydrogen pressure of 20 bar and a reaction duration of 4 h.

These studies provide a collective illustration of advances in catalyst design and operating parameters that significantly influence the efficiency and outcomes of the HDO process. By optimizing these factors, researchers can improve the conversion of crude bio-oils into fuels of higher quality, utility, and economic value.

## 10. Catalyst Deactivation

In the hydrodeoxygenation (HDO) process, catalyst deactivation poses a considerable challenge, mainly due to mechanisms such as carbonaceous deposition, sintering, and the poisoning of catalytic sites. These phenomena affect the performance of HDO catalysts by reducing their effective surface area and altering their chemical functionality. In particular, the presence of nitrogen-, sulfur- and phosphorus-containing compounds from the biomass, together with the role of inherent and reaction produced water in the bio-oil, contribute to catalyst instability at high temperatures [124]. These types of deactivations will be developed further in the following sections.

### 10.1. Deactivation Due to Coking

Coke formation occurs as a result of the polymerization of unsaturated hydrocarbons and the polycondensation of oxygenated organic compounds, causing coke deposits to form on the active sites of the catalyst and blocking the pores. This phenomenon is particularly problematic for materials with small pores, such as zeolites, which limit access to the reactants. With their strong interactions with active metal sites and oxide supports, phenolic compounds play an important role as precursors to coke formation [88,93,103].

The catalyst nature, types of bio-oil compounds or model compounds [150], and obtained intermediates, as well as reaction conditions such as temperature, pressure, and time, considerably influence coke formation. Additionally, compounds containing more than one oxygen atom greatly contribute to coke formation due to their propensity for self-polymerization [150]. For example, He et al. [115] reported catalyst deactivation due to coke formation in the HDO of anisole over Ni-Mo/SiO_2_ at 410 °C and 0.1 MPa, where the high temperatures led to the production of undesirable by-products such as biphenyl and anthracene, the main coke precursors.

**Figure 24:**
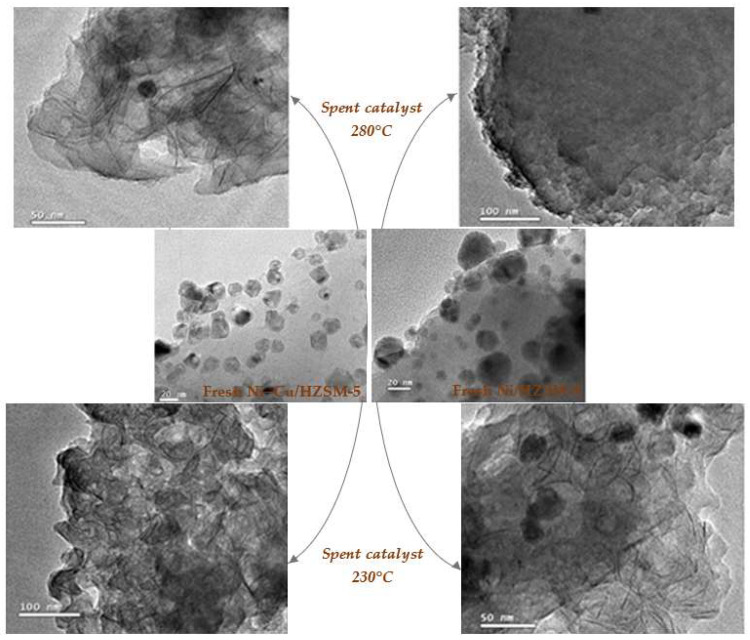
TEM images of fresh and spent catalysts at different reaction temperatures, available from [151].

Additionally, both the Lewis and Brønsted acid sites of zeolite-supported catalysts are susceptible to deactivation by coke formation. The strong acid sites and the confined porous structure of these catalysts promote conditions propitious for coke deposition. Li et al. [151] examined the mechanisms of coke formation on two specific catalysts, Ni/HZSM-5 and Ni-Cu/HZSM-5 (Figure 24), during the HDO of lignin-derived bio-oil at 250 to 330 °C under 2 MPa hydrogen pressure. The authors reported a drastic reduction in catalytic performance due to coke formation on the catalyst surfaces, attributed to the high acidity of the HZSM-5 zeolite. Under this environment, Lewis acid sites rapidly became capped with oxygenated hydrocarbons, while Brønsted acid sites donated protons to these oxygenates, facilitating the formation of carbocations, precursors of soluble coke according to [152].

Additionally, operating conditions such as temperature, H_2_ pressure, and contact time, along with post-reaction treatments like calcination and reduction, significantly influence coke formation during hydroprocessing (Figure 4). For instance, low H_2_ pressure and reaction temperature are known to predispose the process towards coke formation rather than fostering the desired hydrogenation reactions. Li et al. [153] investigated the effect of reaction temperature on coke formation, finding that elevated temperatures resulted in severe coke formation despite improvements in HDO efficiency.

Furthermore, previous treatments such as calcination and reduction can regenerate the spent catalyst, restoring its activity almost to the levels of a fresh catalyst. In their study, Zhu et al. investigated coke formation using a Pt/H-Beta catalyst during the HDO of anisole at 400 °C and atmospheric pressure. They revealed that the acidic nature of the catalyst promoted coke formation; however, the addition of metal to the acidic zeolite improved the catalyst stability and moderately reduced coke formation due to its strong hydrogenation activity [67]. Additionally, maintaining a high hydrogen partial pressure during HDO has been shown to reduce coke formation [154].

Infantes-Molina et al. [155] investigated a comparative study on coke formation between a cobalt phosphide catalyst and a nickel phosphide catalyst during the HDO of dibenzofuran DBF. Their findings showed that although both catalysts possessed high acid sites densities, Ni_2_P catalyst showed both a greater HDO activity and fewer deactivation attributed to a higher quantity of weak acidic sites. In contrast, in the cobalt phosphide catalyst, the substantial presence of strongly acidic sites caused severe coke formation, reducing its HDO activity. This comparative analysis emphasises the significant impact of the acid strength of the catalysts on the degree of coke formation, underscoring the importance of carefully selecting and designing catalysts to minimise deactivation and maximise process efficiency in hydrodeoxygenation operations.

### 10.2. Deactivation Due to Sintering

Sintering or thermal aging of the catalyst is a critical phenomenon that occurs as the reaction temperature increases due to the exothermic nature of HDO process. This process is characterized by the loss of catalytic surface area resulting from the growth of crystallites, pore collapses inside these crystallites, or the degradation and structural collapse of the support material [63,150]. Although not the main focus of much research, notable observations have been made on the catalytic performance evolution under conditions that favor sintering.

Ni catalysts supported on acidic supports, such as transition metal oxides (Al_2_O_3_, TiO_2_), exhibit higher thermal stability compared to those supported on non-acidic or weak acidic supports such as SiO_2_ [118]. This enhanced stability is attributed to the interactions between Ni and the support material. Lan et al. [120] explored the sintering of active Ni on Ni_2_P/SiO_2_ during HDO of guaiacol at 300 °C; they observed a significant reduction in catalytic activity, where conversion rates decreased from 100% to 10% in as little as 8 h. This drastic loss underscores the susceptibility of certain catalysts to thermal degradation under HDO conditions.

Additionally, Ni-doped mesoporous supports, such as Al-SBA-15, Ti-SBA-15, Al-MCM-41, and Ti-MCM-41, provide a dual advantage that mitigates some sintering effects: the enhancement of Ni-support interactions and the confinement offered by the pore size, which limit the crystallite and atom migration and reduce the rate of crystallite growth as they approach the pore diameters [63]. These characteristics are essential for maintaining the structural integrity and activity of Ni-based catalysts subjected to thermal stress during HDO, illustrating the importance of selecting suitable support materials to improve catalyst longevity and efficiency.

### 10.3. Deactivation Due to Poisoning

The poisoning of active sites on the catalyst surface is a key factor in catalyst performance and is influenced by the adsorption strength of chemical species competing with the reactant for these sites [63]. Poisoning occurs through the strong chemisorption of various chemical species—including reagents, intermediates, products, and impurities—on the catalytic sites [156]. In lignin-derived bio-oil HDO, common poisons include oxygen-containing compounds such as water and CO. In particular, water, which is produced during the HDO process, can significantly reduce the efficiency of the catalyst by competing with the reagents for adsorption on the active sites of the catalyst [157].

Recent studies have revealed the detrimental effects of water poisoning on catalytic performance. Water not only adsorbs on active sites but can also alter the chemical structures of these sites, leading to a decreased catalytic activity. For instance, Li et al. [116] investigated the HDO of anisole at 300 °C and 1.5 MPa using NiP/SiO_2_ and NiMoP/SiO_2_ catalysts and revealed that the water generated as a by-product led to the formation of nickel metal oxides and phosphate oxides (resulting from the oxidation of NiP), which were significantly less active than the original phosphide forms. In addition, Mortensen et al. [158] also reported that in their phenol and octanol HDO deactivation studies, water caused the deactivation of Ni-MoS_2_/ZrO_2_ catalysts by undergoing competitive adsorption on the active sites, which facilitated the conversion of sulfide to sulfate at the edges of the MoS_2_ catalyst.

## 11. Hydrodeoxygenation Set-Up

**Figure 25:**
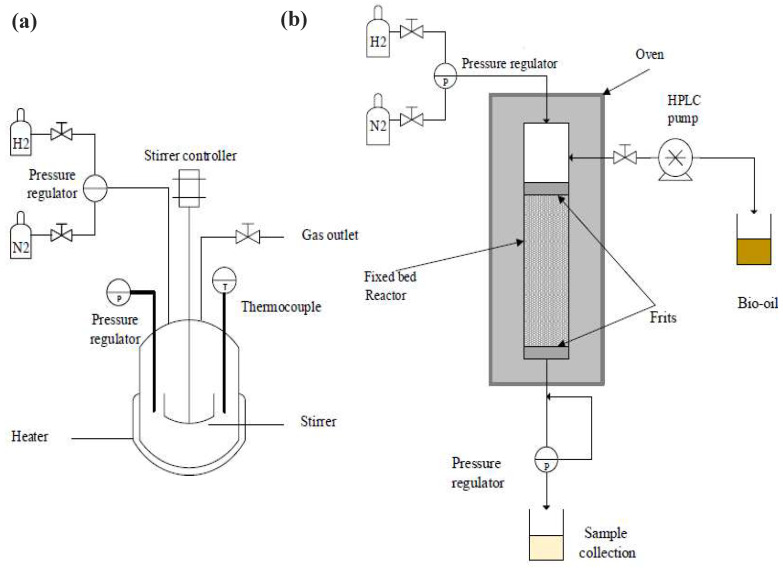
Schematic diagram of the most common reactors used in the HDO; batch reactor (**a**) and continuous flow reactor (**b**), adapted from [159,161].

The HDO process is typically carried out at reaction temperatures between 250 and 500 °C [88,159,160] and high hydrogen pressures in various reactor configurations, such as batch reactors in the liquid phase (Figure 25a) and continuous flow fixed-bed reactors in the gas phase operating between 5 and 50 MPa [126,159]. And in the case of a continuous reactor, the liquid hourly space velocity values (LHSV) are between 0.05 and 2 h^−1^. For the reaction parameters in both the batch and continuous set-up, the temperature and pressure were generally proved to be key factors in governing the final oxygen content of the upgraded bio-oil.

Bukhtiyarova et al. [159] highlighted the fundamental disparities between batch and continuous flow reactors, outlining their distinctive operating characteristics and their implications for catalytic processes. A batch reactor, depicted in Figure 25a, functions as a transitory reactor in which the reaction mixture and catalyst are loaded and subsequently submitted to high-temperature and high-pressure conditions. Contrarily, a continuous flow reactor (Figure 25b) functions as a steady-state reactor, in which reactants are continuously fed into the reactor entrance and traverse the catalyst bed. Particularly, after the reaction, the unreacted reactant/product mixture exits the reactor outlet, distinguishing it from the batch reactor. The comparison between batch and continuous flow reactors underscores crucial differences in their operational dynamics and implications for catalytic processes.

Usually, researchers like [89,159,162] advocate for conducting reactions in continuous flow reactors due to numerous advantages over batch reactors, such as better mixing of reagents, superior interfacial mass and energy transfer characteristics, reduced operational costs, mitigation of byproduct generation through enhanced control over reaction parameters, simplified scalability, and heightened process safety. When applied to the HDO of bio-oil, continuous reactors have demonstrated distinct characteristics, including a tendency towards relatively higher temperatures and H_2_ consumption. Despite these variances, continuous reactors exhibit a propensity for achieving elevated DOD rates, primarily attributed to the generation H_2_O as a byproduct during the reaction.

Additionally, continuous reactors typically yield lower levels of coke formation, indicating their potential to deliver more sustainable and efficient bio-oil upgrading processes. Consequently, it is plausible that the utilization of continuous reactors may lead to the production of superior quality upgraded bio-oil and sustain prolonged catalyst activity. Leveraging the benefits conferred by continuous reactors holds promise for optimizing the efficacy and sustainability of bio-oil refining technologies, thereby contributing to the advancement of renewable energy solutions.

## 12. Conclusions

Biomass, derived from plants or animals, undergoes fast pyrolysis to yield pyrolytic bio-oil, containing 30–40 wt% organic chemicals and 25 wt% water. Challenges like high water content, acidity, oxygen proportion, and low calorific value hinder direct use. Hydrodeoxygenation (HDO) is crucial as it saturates aromatic components and alkenes, raising the bio-oil’s calorific value by enhancing the H/C ratio and reducing the O/C ratio, removing oxygen as water. Despite advancements, biomass pyrolysis oils require further research, catalyst optimization, and understanding of complex reactions routes (decarbonylation, hydrogenation, cracking, hydrocracking) that play a crucial role for determining the quality and selectivity of the final upgraded bio-oil products before practical applications. Successful HDO hinges on suitable catalysts and model compounds, pivotal for enhancing biofuel properties and advancing sustainable energy solutions.

Catalytic carriers are essential for HDO, facilitating active phase dispersion and providing essential functional sites for effective deoxygenation reactions. Various catalyst types, including sulfides (e.g., CoMoS_2_, NiMoS_2_), oxides (e.g., MoO_x_, NiO_x_), carbides (e.g., Mo_2_C), phosphides (e.g., MoP), and noble metals (e.g., Pt, Pd), have been assessed for HDO efficacy. Sulfide catalysts operate via electron transfer, necessitating sulfur sources for optimal performance, while oxide and bimetallic catalysts like Pt-WO_x_/Al_2_O_3_ use acid sites to enhance activity. Transition metals such as Pt, Pd, and Ni offer sulfur-free pathways but are susceptible to sulfur contamination. Ni_2_P distinguishes itself with high electronic conductivity and chemical durability, eliminating the need for additional sulfur sources. Phosphorus in Ni_2_P enhances hydrogenation and fosters Brønsted acid sites, crucial for efficient deoxygenation, showing superior performance in converting bio-oil model compounds with high selectivity and stability. Nickel-modified catalysts, especially Ni_2_P on supports like ZSM-5 or SBA-15, demonstrate enhanced efficiency and stability, highlighting Ni_2_P’s role in advancing HDO technology.

The HDO of bio-oil involves complex reaction pathways, which researchers are attempting to clarify using model compounds representing key constituents of bio-oil. Studies on phenolic compounds such as guaiacol have revealed various pathways, such as hydrogenation and deoxygenation, that influence the selectivity of products, such as cycloalkanes or arenes. Research on aldehydes such as furfural illustrates multiple pathways, such as hydrogenolysis and decarbonylation, which influence products such as 2-methylfuran. Carboxylic acids, such as acetic acid, present pathways such as ketonization and hydrogenolysis, where catalyst conditions affect the distribution of products. Mixtures present additional challenges; for example, hydroxyl-containing molecules influence pathways and selectivity, while combinations such as guaiacol and propionic acid show potential for simultaneous hydrodeoxygenation and esterification. Catalyst design and understanding of reaction mechanisms are crucial for optimizing HDO, where reactor conditions such as temperature and pressure improve conversion rates and selectivity, while acidity facilitates reactions such as esterification and hydrolysis. Higher temperatures favor certain reactions like hydrogenation, while higher hydrogen pressures enhance hydrogenation processes, leading to improved conversion efficiencies.

In HDO, catalyst deactivation involves coking, sintering, and poisoning of the catalytic sites. Coking is a consequence of hydrocarbon polymerization, which is influenced by the type of catalyst, bio-oil composition, and conditions. Catalysts supported by zeolite are susceptible due to the strong acid sites and their confined structure. Sintering reduces the surface area at higher temperatures, which is achieved with Ni doped mesoporous supports. Poisoning by species such as water and CO decreases catalytic efficiency and alters active site structures. Coking dominates, requiring adaptation of the catalyst design and operations to mitigate its impact. HDO challenges include gas–liquid-phase equilibrium at elevated temperatures and pressures, accelerated coke formation by highly acidic catalysts, and the influence of pore size on reaction rate. Bio-oil viscosity poses equipment problems, catalyst recycling remains problematic, and L-glucose appears as a coke precursor. These complexities require further research to optimize HDO processes.

A comparison between batch and continuous flow reactors highlights the operational differences affecting the catalytic processes. Continuous flow reactors offer advantages such as improved mixing, parameter control, and increased safety, achieving higher HDO rates and reduced coke formation compared to batch systems. Continuous reactors promise sustainable upgrading of bio-oil, leveraging prolonged catalyst activity and efficiency to advance renewable energy solutions.

## Figures and Tables

**Table 1:** Typical properties of pine wood, bio-oil from fast pyrolysis and fossil-based oil, adapted from [18,19,20,21].

Physical Properties		Pine Wood Bio-Oil	Fast Pyrolysis Bio-Oil	Fossil Petroleum
Moisture content (wt%)		15–30	15–30	0.1
pH		-	2–3.7	-
Specific gravity		-	1.2	0.94
Elemental analysis (wt%)	C	49	54–58	83–86
	H	6	5.5–7.0	11
	O	44	35–40	1
	N	0.06	0–0.2	0.3
	Ash	0.3	0–0.2	0.1
High heating value (HHV) (MJ/kg)		20	16–19	40
Viscosity (cP at 50 °C)		-	40–100	180
Solid content (wt%)		-	0.2–1	1

**Table 2:** Advantages and disadvantages of various upgrading technologies currently developed for upgrading of pyrolysis bio-oil.

Upgrading Methods	Objectives	Advantages	Disadvantages	Reference
Emulsification	Enhance the miscibility of bio-oils with diesel fuel Use bio-oils in combustion engines	Simple operation steps	High energy input High cost of surfactant Corrosion problems	[59]
Solvent Addition	Reduce the aging effect: alcohol: methanol, ethanol, and isopropanol are used.	Easy operation and increases in bio-oil’s lower heating value, reduces density and viscosity	Decrease in the flashpoint of bio-oils; Unfavorable materials cannot be removed (oxygen)	[40,60]
Steam reforming	Hydrogen production from bio-oil reforming	High yield Better regeneration of the catalyst	Costly Fully developed reactors High operating temperature	[45]
Hydrotreatment (HDO)	Removal of sulfur, nitrogen, and oxygen heteroatom	Utilizing compressed hydrogen to remove oxygen, increasing heating value and lowering bio-crude oil viscosity, moderate reaction condition	Harsh conditions, complicated equipment, easy reactor blockage, and catalyst deactivation	[26,61,62,63]
Esterification	Organic acids (from acid, acetic acid, propionic acid, etc.) in bio-oil can be converted to their corresponding esters.	The most practical approach (simplicity, the low cost of some solvents, and their beneficial effects on the oil properties)	Low oil production and poor performance	[55]
Catalytic cracking	Break down larger hydrocarbon molecules into smaller hydrocarbon molecules, often involving subsequent hydrogenation.	Makes large quantities of light products. High yield of light products	High cost, harsh, hydrogen consumption High pressure-resistant reactor required Catalyst deactivation, reactor clogging	[46,60]
Supercritical fluid	Obtain high yields and qualities of the bio-oil. Some organic solvents, such as ethanol, methanol, water and CO_2_ are used/	Higher oil yield, better fuel quality (lower oxygen content, lower viscosity)	High cost of solvent High-pressure resistant reactor required	[56]

**Table 3:** HDOs of different compounds over sulfide catalysts.

Catalyst	Oxygenated Compound	Deoxygenated Compound	Reference
NiMoS	Guaiacol	Phenol, Catechol, Cyclohexane	[36,70]
MoS_2_	Phenol	Benzene	[71]
NiM@C	Guaiacol	Cyclohexanol, Phenol, Cyclohexane	[61]
CoMoZ	Anisole	Benzene, Toluene, Xylene	[65]
CoMoS/Al_2_O_3_	Guaiacol	Cyclohexene, Cyclohexane, Benzene	[72]
CoMoS	*P*-cresol	Toluene, Methylcyclohexane, 3-methylcyclohexene	[73]
Ni-Mo	4-methylphenol	Toluene, Methylcyclohexane, and 3–4 methylcyclohexene	[74]
NiMo/SBA-15	Guaiacol	Benzene, Myclohexene, Cyclohexane, Phenol	[75]
NiMoP/HMS	Guaiacol	Biphenyl, Clohexylbenzene, Dicyclohexyl, Tetrahydrodibenzothiophene	[76]
Co–Mo–P/MgO	Phenol	Benzene, Cyclohexylbenzene, Cyclhexylphenol	[77]

## Abbreviations

ALCC	Aldol crossed condensation
ALSC	Aldol Selt-condensation
BXT	Benzene, Xylene, Toluene
CGE	Carbon gasification efficiency
CSO	Camelina oil
DBF	Dibenzofuran
DDO	Direct deoxygenation
DHO	Dehydroxylation
DHY	Dehydration
DOD	Degree deoxygenation
DME	Demethylation
DMO	Demethoxylation
EEO	Ether extracted bio-oil
FAME	Fatty acid methyl ester
FPBO	Beech wood fast pyrolysis-oil
HBA	Hydrogen bond acceptor
HBD	Hydrogen bond donor
HDO	Hydrodeoxygenation
HDN	Hydrodenitrogenation
HGD	Hydrogenolysis
HGE	Hydrogen gasification efficiency
HHV	High heating value
HLB	Hydrophilic-lipophilic balance
HMF	5-(hydroxymethyl)furfural
HMTHFA	5-(hydroxymethyl)tetrahydrofuran-2-carbaldehyde
HYD	Hydrogenation
LHV	Low heating value
MCH	Methylcyclohexane
MSW	Municipal solid waste
Ni_2_P	Nickel phosphide
OVOCs	Oxygen-containing volatile organic compounds
SCF	Supercritical fluid
SR	Steam reforming
TAN	Total acid number
THF	Tetrahydrofuran
TPR	Temperature-programmed reaction
WHSV	Weight hourly space velocity
WGS	Water gas shift
TEM	Transmission electron microscopy
